# Advances in the development of amorphous solid dispersions: The role of polymeric carriers

**DOI:** 10.1016/j.ajps.2023.100834

**Published:** 2023-08-01

**Authors:** Jie Zhang, Minshan Guo, Minqian Luo, Ting Cai

**Affiliations:** a Department of Pharmaceutics, School of Pharmacy, China Pharmaceutical University, Nanjing 211198, China; b Department of Pharmaceutical Engineering, School of Engineering, China Pharmaceutical University, Nanjing 211198, China; c College of Biological and Chemical Engineering, Changsha University, Changsha 410022, China

**Keywords:** Amorphous solid dispersions, Polymeric carriers, Stability, Dissolution, Bioavailbility, Molecular interactions

## Abstract

Amorphous solid dispersion (ASD) is one of the most effective approaches for delivering poorly soluble drugs. In ASDs, polymeric materials serve as the carriers in which the drugs are dispersed at the molecular level. To prepare the solid dispersions, there are many polymers with various physicochemical and thermochemical characteristics available for use in ASD formulations. Polymer selection is of great importance because it influences the stability, solubility and dissolution rates, manufacturing process, and bioavailability of the ASD. This review article provides a comprehensive overview of ASDs from the perspectives of physicochemical characteristics of polymers, formulation designs and preparation methods. Furthermore, considerations of safety and regulatory requirements along with the studies recommended for characterizing and evaluating polymeric carriers are briefly discussed.

## Graphical abstract

**Figure:**
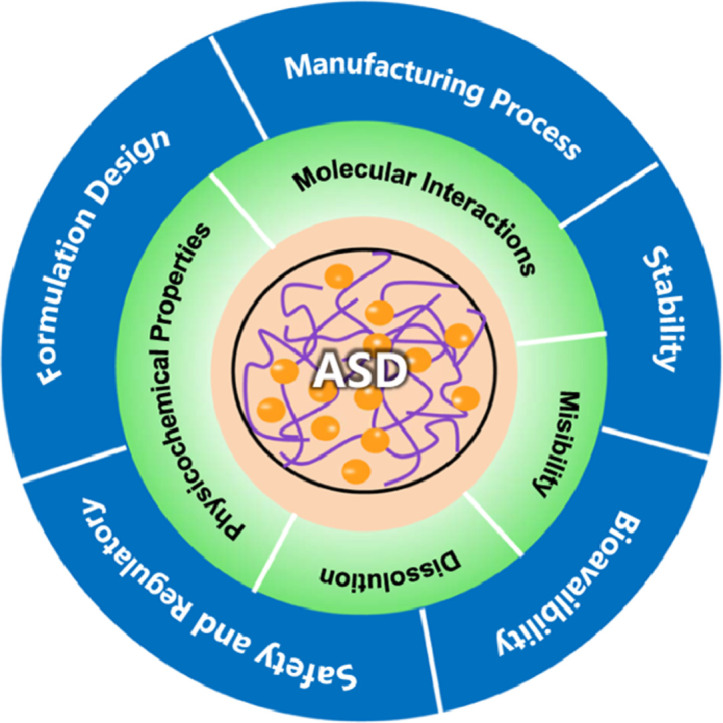

## 1 Introduction

Amorphization is an effective approach for the development of poorly soluble drugs contained in biopharmaceutical classification system (BCS) class II. The conversion of crystalline pharmaceuticals to amorphous counterparts can increase solubility, dissolution rates and bioavailability for poorly soluble drugs [1], [2], [3], [4], [5]. However, since the amorphous drugs are thermodynamically unstable, they tend to recrystallize during manufacturing, storage, and use of the products. To overcome this problem, polymeric carriers have been widely used to stabilize amorphous drugs by forming amorphous solid dispersions (ASDs), which are considered one of the most effective approaches to deliver poorly soluble drugs. The ASD products on the market are summarized in Table 1 [2,6].

**Table 1:** Examples of commercial ASD products.

Trade name	Manufacturer	Drug	Processing technology	Polymer	Dosage form	Year of FDA approval
Cesamet^Ⓡ^	Valeant	Nabilone	Solvent evaporation	PVP	Tablet	1985
Isoptin^Ⓡ^	Abbott	Verapamil	HME(holt melt extrusion)	HPC/HPMC	Tablet	1987
Sporanox^Ⓡ^	Janssen	Itraconazole	Fluid-bed bead layering	HPMC	Capsule	1992
Prograf^Ⓡ^	Fujisawa	Tacrolimus	Solvent evaporation	HPMC	Capsule	1994
Kaletra^Ⓡ^	Abbott	Ritonavir/ Lopinavir	HME	PVP/VA64	Tablet	2007
Intelence^Ⓡ^	Janssen	Etravirine	Spray drying	HPMC	Tablet	2008
Samsca^Ⓡ^	Otsuka	Tolvaptan	Spray drying	HPC	Tablet	2009
Zortress^Ⓡ^	Novartis	Everolimus	Spray drying	HPMC	Tablet	2010
Norvir^Ⓡ^	Abott	Ritonavir	HME	PVP/VA64	Tablet	2010
Onmel^Ⓡ^	Stiefel	Itraconazole	HME	HPMC	Tablet	2010
Zelboraf^Ⓡ^	Roche	Vemurafenib	Solvent controlled precipitation	HPMCAS	Tablet	2011
Incivek^Ⓡ^	Vertex	Telaprevir	Spray drying	HPMCAS	Tablet	2011
Kalydeco^Ⓡ^	Vertex	Ivacaftor	Spray drying	HPMCAS	Tablet	2012
Noxafil^Ⓡ^	Merck	Posaconazole	HME	HPMCAS	Tablet	2013
Astagraf XL^Ⓡ^	Astellas Pharma	Tacrolimus	Wet granulation	HPMC;EC	Capsule	2013
Belsomra^Ⓡ^	Merck	Suvorexant	HME	PVP/VA64	Tablet	2014
Harvoni^Ⓡ^	Gilead Sciences	Ledipasvir/Sofosbuvir	Spray drying	PVP/VA64	Tablet	2014
Viekira XR™	AbbVie	Dasabuvir/Ombitasvir/Paritaprevir/Ritonavir	HME	PVP/VA64;HPMC	Tablet	2014
Epclusa^Ⓡ^	Gilead Sciences	Sofosbuvir/Velpatasvir	Spray drying	PVP/VA64	Tablet	2016
Orkambi^Ⓡ^	Vertex	Lumacaftor/Ivacaftor	Spray drying	HPMCAS	Tablet; Granule	2016
Venclexta™	AbbVie	Venetoclax	HME	PVP/VA64	Tablet	2016
Zepatier^Ⓡ^	Merck	Elbasvir/Grazoprevir	Spray drying	PVP/VA64	Tablet	2016
Mavyret™	AbbVie	Glecaprevir/Pibrentasvir	HME	PVP/VA64	Tablet	2017
Vosevi^Ⓡ^	Gilead Sciences	Sofosbuvir/Velpatasvir /Voxilaprevir	Spray drying	PVP/VA64	Tablet	2017
Erleada™	Janssen	Apalutamide	Spray drying	HPMCAS	Tablet	2018
Symdeko^Ⓡ^	Vertex	Tezacaftor/ivacaftor+ivacaftor	Spray drying	HPMCAS	Tablet	2018
Braftovi^Ⓡ^	Array	Encorafenib	HME	PVP/VA64	Capsule	2018
Trikafta™	Vertex	Elexacaftor/ivacaftor/tezacaftor	Spray drying	HPMCAS	Tablet	2019
Tukysa™	Seagen	Tucatinib	Spray drying	PVP/VA64	Tablet	2020

Polymeric materials generate ASDs and provide many benefits [7]. The polymers in ASDs can often increase the physical stabilities of amorphous drugs by inhibiting crystallization [7], [8], [9], [10], [11], [12], [13]. In addition, the presence of hydrophilic carriers leads to improved wettability of the amorphous drugs [7]. Furthermore, the appropriate polymeric carriers also improve the bioavailability by inhibiting drug precipitation in the gastrointestinal tract [7,^14^,15].

The selection of polymer carriers impacts the manufacturing processes, stabilities, solubilities and bioavailabilities of the ASDs. Thus, the selection of a polymer is the key factor determining the success of ASD development. Polymers typically used in forming ASDs include polyvinyllactam polymers such as polyvinylpyrrolidone (PVP), polyvinylpyrrolidone-vinyl acetate copolymer (PVP/VA) and Soluplus^Ⓡ^, cellulose derivatives such as hydroxypropyl cellulose (HPC), hydroxypropyl methylcellulose (HPMC), hydroxy ethyl cellulose, hypromellose acetate succinate (HPMCAS), hydroxypropyl methylcellulose phthalate (HPMCP), cellulose acetate phthalate (CAP) and polymethacrylates (Eudragit^Ⓡ^ E, L, S) [4,16]. These polymers have different physicochemical properties, including glass transition temperatures (*T*_g_), hygroscopicities, degradation temperatures and solubilization capacities, which may result in the distinct capabilities of the ASDs.

There are many excellent reviews introducing the ASDs including aspects of physical stability, preparation methods, characterization, mechanism on bioavailability, etc. [1], [2], [3], [4], [5],[17], [18], [19], [20]. Despite the critical roles of polymers in forming ASDs, selecting an appropriate polymer is usually by trial-and-error process [16]. This review is aimed to address the physicochemical properties and roles of polymers in the formulation designs and manufacturing processes of ASDs. In addition, the regulatory environment for polymeric excipients used in ASDs is discussed.

## 2 Physicochemical properties of the polymeric carriers used in ASDs

**Fig. 1:**
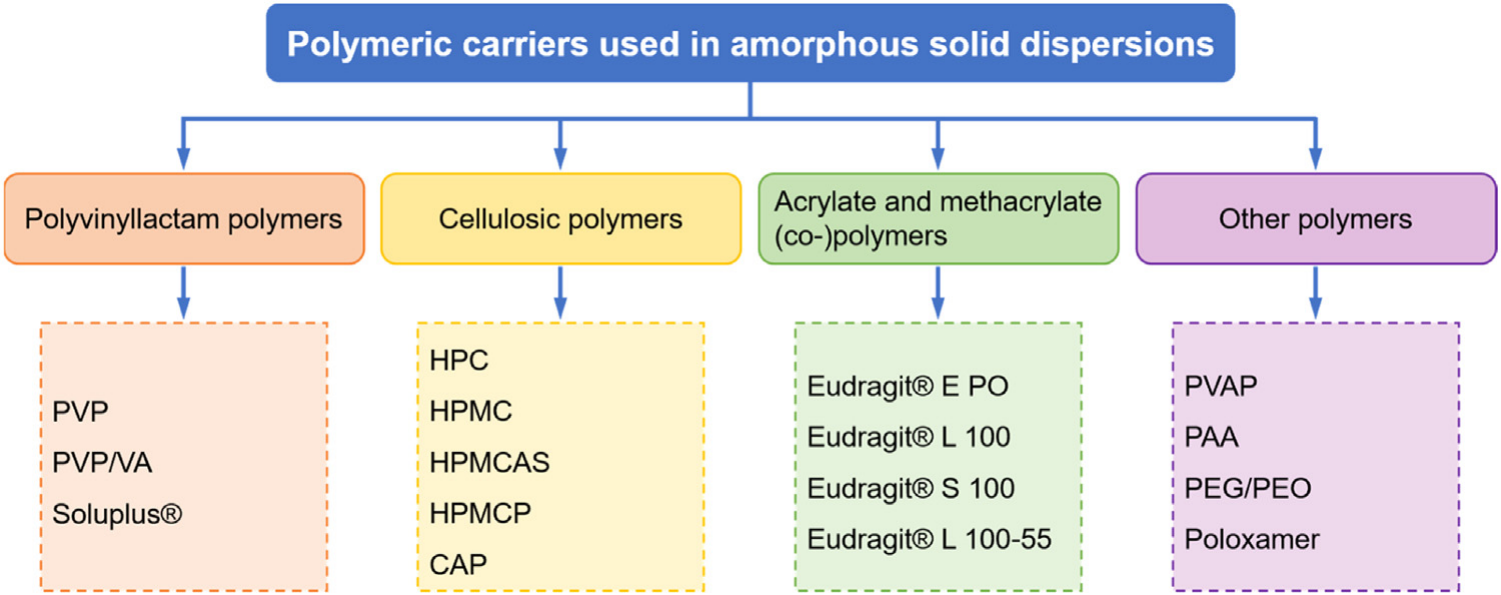
Classification of polymeric carriers based on their chemical structures.

A variety of polymers have been utilized in ASD formulations. Those polymeric carriers can be classified into different groups according to their chemical structures: polyvinyl lactam polymers, cellulosic polymers, acrylate and methacrylate (co-)polymers, and some other types (Fig. 1). Tables 2 and 3 summarize the physicochemical properties of the different types and grades of polymers used in ASDs, including solubility, *T*_g_, hygroscopicity, and degradation temperature. The most commonly used polymeric carriers in ASDs are discussed in the following sections.

**Table 2:** Structure and key physical properties of polymeric carriers [14,^15^,[21], [22], [23], [24].

Chemical category and classification	Chemical name	Abbreviation	Chemical structure	Key physical attributes
Polyvinyllactam polymers	Polyvinylpyrrolidone	PVP		• High hygroscopicity • Different grades based on average molecular weight
	Copovidone	PVP/VA		• High degradation temperature • Medium hygroscopicity
	Polyvinylcaprolactam–polyvinyl acetate–polyethyelne glycol graft copolymer	Soluplus^Ⓡ^		• Amphiphilic structure • Low *T*_g_ • High degradation temperature • Low hygroscopicity
Cellulosic polymers	Hydroxypropyl methyl cellulose	HPMC		• Low hygroscopicity • Different grades based on average molecular weight
	Hydroxypropyl cellulose	HPC		• Low hygroscopicity • Low *T*_g_
	Hypromellose acetate succinate	HPMCAS		• Modified from HPMC by esterification with acetic acid anhydride and succinic acid anhydride. • Insoluble below pH 5.0
	Hydroxypropyl methylcellulose phthalate	HPMCP		• Has a phthalic acid group • Enteric coating material
	Cellulose acetate phthalate	CAP		• Dissolves at higher pH (more than 6) • Has a phthalic acid group • Enteric coating material
Acrylate and methacrylate (co-)polymers	Poly(butyl methacrylate-co-(2-dimethylaminoethyl) methacrylate-comethyl methacrylate) 1:2:1	Eudragit^Ⓡ^ E PO		• Solubilizes at pH below 5.5 • Low hygroscopicity
	Hetero block co-polymers of poly(methacrylic acid- co-methyl methacrylate)1:1	Eudragit^Ⓡ^ L 100		• Ionizes above pH 6.0 • Enteric coating material
	Hetero block co-polymers of poly(methacrylic acid- co-methyl methacrylate)1:2	Eudragit^Ⓡ^ S 100		• Ionizes above pH 7.0 • Enteric coating material
	Poly(methacrylic acid-co-ethyl acrylate) 1:1	Eudragit^Ⓡ^ L 100–55		• Ionizes above pH 5.5 • Enteric coating material
Other types of polymers	Polyvinyl acetate phthalate	PVAP		• Solubilizes at pH above 5.0 • Has a phthalic acid group
	Poly(acrylic acid)	PAA		• pKa=4.5 • Has a high density of carboxylic acid groups
	Polyethylene glycol /polyethylene oxide	PEG/PEO		• Water soluble • Low *T*_g_
	Poly- (ethylene oxide)−poly(propylene oxide)−poly(ethylene oxide) triblock copolymers	Poloxamer		• Water soluble • Low *T*_g_

**Table 3:** Physicochemical properties of different types and grades of polymers used in ASD [14,^15^,[21], [22], [23], [24].

Category	Chemical name	Polymer type	Molecular weight	Solubility	Hygroscopicity	*T*_g_ (or *T*_m_ °C)	Degradation temperature ( °C)
Polyvinyllactam polymers	PVP	Kollidon^Ⓡ^ 12 PF	2,000 – 3000	Water soluble	High	72	196
		Kollidon^Ⓡ^ 17 PF	7,000 – 11,000			140	217
		Kollidon^Ⓡ^ 25	28,000 – 34,000			153	166
		Kollidon^Ⓡ^ 30	44,000 – 54,000			160	171
		Kollidon^Ⓡ^ 90F	1,000,000 – 1500,000			177	194
	PVP/VA	Kollidon^Ⓡ^ VA64	MW 45,000 – 70,000	Water soluble	High	105	270
	Soluplus^Ⓡ^	Soluplus^Ⓡ^	MW 90,000 – 140,000	Water soluble	High	72	278
Cellulosic polymers	HPMC	Pharmacoat^Ⓡ^ 606	MW – 10,000	Water soluble	High	139	244
		Methocel™ K100LV	MW – 25,000			147, 168	259
		Methocel™ K100M	MW – 150,000			96, 173	259
	HPC	Klucel^Ⓡ^ LF	MW – 95,000	Water soluble	High	0[25]	170–200
	HPMCAS	Shin-etsu AQOAT^Ⓡ^ MF	MW – 18,000 (3 cps)	Dissolves above pH 5.0	Low	122	204
	HPMCP	HP-55	MW – 45,600 (40 cps)	Dissolves above pH 5.0	Low	147	194
		HP-50	MW – 37,900 (55 cps)			143	199
	CAP	N/A	MW – 2534.12	Dissolves at higher pH (more than 6)	Low	175	200
Acrylate and methacrylate (co-)polymers	Eudragit^Ⓡ^ E PO	N/A	MW – 47,000	Dissolves in gastric fluid below pH 5.0	Low	52	250
	Eudragit^Ⓡ^ L 100	N/A	MW – 125,000	Dissolves above pH 6.0	Low	195	176
	Eudragit^Ⓡ^ S 100	N/A	MW – 125,000	Dissolves above pH 7.0	Low	173	173
	Eudragit^Ⓡ^ L 100–55	N/A	MW – 320,000	Dissolves above pH 5.5	Low	111	176
Other polymers	PVAP	N/A	MW 47,000–60,700	Dissolves above pH 5	Low	42.5	150
	PAA	N/A	MW 1,800–450,000	Water soluble	Low	126	200
	PEG/PEO	N/A	MW 1,000–7,000,000	Water soluble	Low	*T_m_=55–66*	>200
	Poloxamer	N/A	MW 7,600–17,400	Water soluble	Low	*T_m_=52–57*	>200

### 2.1 Polyvinyllactam polymers

Polyvinyllactam polymers are synthesized from vinylpyrrolidone or vinylcaprolactam monomers [14,15]. The vinyllactam can form the homopolymer PVP or combine with vinyl acetate to form Copovidone (PVP/VA) [14,15]. Moreover, Soluplus^Ⓡ^, a copolymer containing polyvinyl caprolactam, polyvinyl acetate (PVAc), and PEG, has been increasingly used in the formulations of ASDs [14].

#### 2.1.1 Polyvinylpyrrolidone (PVP)

PVP is a common polymeric carriers applied to produce ASDs [14,15]. The MWs of PVP range from 2500 to 3,000,000 Da [14,15]. The K-value is used to indicate the average degree of polymerization [14,15]. The K-values of PVP are usually part of the trade name, with values ranging from 12 to 120 [14,^15^,26].

The ASDs prepared by PVP can often induce a higher solubility for a poorly water-soluble drug [27,28]. For instance, an ASD of curcumin containing PVP exhibited complete dissolution of curcumin in 0.1 N HCl within 30 min, whereas crystalline curcumin showed negligible drug release over 90 min [27]. In a study of silymarin ASDs, the PVP K17-based ASD significantly improved the dissolution and bioavailability [28]. In an *in vitro* dissolution test, the PVP K17-based ASD achieved a cumulative drug release of 98% in 15 min, while the release rate of crystalline silymarin was less than 30% after 120 min [28]. *In vivo*, the *AUC* and the *C*_max_ of silymarin ASD enhanced 2.4-fold and 1.9-fold compared to those of the crystalline drug, respectively [28].

**Fig. 2:**
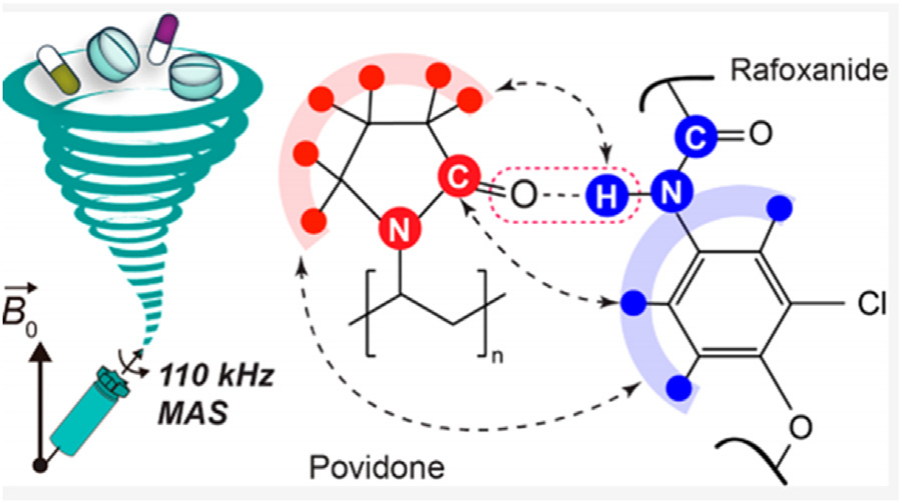
The hydrogen bonding interactions in rafoxanide-PVP ASDs. Adapted from [30] with the permission.

The carbonyl oxygen in PVP (a hydrogen-bond acceptor) forms molecular interactions with drugs containing hydrogen-bond donors. In an early study**,** Taylor and Zografi used IR spectroscopy to reveal hydrogen bonds between the carboxylic acid hydroxyl groups of indomethacin and the amide carbonyl groups of PVP in the ASD [29]. Recently, Li et al. investigated the intramolecular interactions between rafoxanide and PVP in ASDs with solid-state NMR (ssNMR) (Fig. 2) [30]. Intermolecular hydrogen bond between the carbonyl groups of PVP and the amide groups of rafoxanide was confirmed by the chemical shifts of the rafoxanide amide proton and the PVP aliphatic protons [30].

The molecular interactions between PVP and drug molecules can suppress the phase separation and subsequent recrystallization of amorphous drugs [31,32]. Kothari et al. reported that hydrogen bonds were observed between nifedipine and PVP in an ASD, resulting in the slower molecular mobility and crystallization [32]. PVP can disrupt the self-interactions of drugs through formation of drug-polymer interactions [33]. Munson and coworkers used ^13^C solid-state NMR to quantitatively investigate the hydrogen bonding interactions in an ASD containing PVP and indomethacin with a ^13^C-labeled carboxylic acid carbon [33]. They found that the carboxylic acid dimer interactions of the indomethacin molecules were gradually disrupted by increasing the content of PVP in the ASD [33].

The impacts of different PVP molecular weights on the physical stabilities of ASDs have been studied but remain controversial [9,[34], [35], [36], [37], [38]. Mohapatra et al. found that as the PVP molecular weights increased in the ASDs, molecular mobility of indomethacin decreased, and thus, the crystallization inhibitory effect of PVP increased [36]. In a study of bicalutamide and PVP with different K-values (K10, 30, and 90), the ASD containing PVP K90 was the least effective crystallization inhibitor among the polymers investigated [38]. The authors suspected that the ASD comprising PVP K90 exhibited a higher free volume than those prepared from PVP K10 and K30, thereby leading to weaker inhibition [38].

PVP-based ASDs are easily influenced by moisture due to the hydrophilic nature of the PVP. Moisture uptake decreases physical stability of the dispersion by increasing molecular mobility [39], [40], [41], [42], [43]. Duong et al. used environment-sensitive fluorescence probes and monitored the impacts of moisture or bulk water on phase separation in the ASD containing PVP and ritonavir [43].

#### 2.1.2 Copovidone (PVP/VA)

PVP/VA is a vinylpyrrolidone-vinyl acetate copolymer, and the widely used ratio of vinylpyrrolidone to vinyl acetate is 6:4. It is commonly used in manufacturing ASDs due to its excellent processability and low hygroscopicity. PVP/VA64 has a *T*_g_ of approximately 100 °C and a degradation temperature above 230 °C. Due to its excellent stability and processability, it is widely used for manufacturing ASDs by solvent or melting (fusion) methods [44,45].

PVP/VA is relatively less hygroscopic than PVP due to the vinyl acetate component in the copolymer chain. Weuts et al. reported that PVP/VA-based ASDs contained less moisture, and the deviation of *T*_g_ between the experimental and calculated values was much smaller than that of PVP-based ASDs [46]. In another study, a TGA analysis showed that the ASDs comprising PVP-K30 exhibited greater water uptake than ASDs containing PVP/VA upon exposure to high relative humidity [47]. The vinyl acetate in the PVP/VA influenced the drug-polymer interactions. Sarpal et al. observed interactions between felodipine and PVP, PVP/VA and PVAc with ^13^C ssNMR spectra [31]. The strengths of molecular interactions decreased following order PVP > PVP/VA > PVAc [31]. Yuan et al. reported that a large proportion of indomethacin (IMC) molecules exhibited self-interactions involving carboxylic acid cyclic dimers in the amorphous state [33]. PVP and PVP/VA form hydrogen bonds with indomethacin, thus disrupting the drug self-interactions [33]. Compared to PVP, the extent of hydrogen bonding between IMC and PVP/VA was weaker due to the weaker hydrogen bonding capabilities of the vinyl acetate groups [33]. Kestur et al. evaluated the impact of polymer type on the crystallization of felodipine, and they found that PVP/VA was less effective in suppressing crystallization of felodipine than PVP, which was consistent with the strength/extent of intramolecular interactions [48]. It has also been reported that the vinyl acetate content influenced the drug solubility in polymeric carriers. Sun et al. studied the indomethacin and nifedipine solubilities in PVP, PVP/VA and PVAc. Their results showed that the amounts of drugs dissolved in the polymers decreased following order PVP > PVP/VA > PVAc based on different drug-polymer interactions [49].

#### 2.1.3 Polyvinylcaprolactam–polyvinyl acetate–polyethylene glycol graft copolymer (Soluplus^Ⓡ^)

Soluplus^Ⓡ^ is a graft copolymer composed of three monomers, including polyethylene glycol, polyvinyl caprolactam, and polyvinyl acetate. Soluplus^Ⓡ^ is a polymer with a high molecular weight (90,000 to 140,000 g/mol), a relatively low *T*_g_ (72 °C), and a high degradation temperature (above 250 °C) [50], [51], [52], [53].

Since Soluplus^Ⓡ^ is an amphiphilic polymer, it can provide high solid-state solubilization capacities for drugs. Soluplus^Ⓡ^ can form micelles in solution, thereby enhancing the solubilities and bioavailabilities of poorly soluble drugs [54], [55], [56]. Lian et al. reported that a 9-nitrocamptothecin ASD containing Soluplus^Ⓡ^ enhanced the oral bioavailability [57]. Metre et al. found that the apparent solubility of rivaroxaban in Soluplus^Ⓡ^-based ASDs was significantly improved due to the formation of micelles during dissolution [58]. The pharmacokinetic study also showed that Soluplus^Ⓡ^-based ASDs exhibited better *in vivo* performance than ASDs using other polymers [58].

Soluplus^Ⓡ^ is less hygroscopic than PVP and PVP/VA. Caron et al. reported that the sulfonamide ASDs prepared with Soluplus^Ⓡ^ remained dry and powdery during the condition of 60% RH/25 °C, while the PVP-based ASD turned into a sticky paste [59]. TGA analyses showed a 3.4% moisture loss for the sulfadimidine/Soluplus^Ⓡ^ ASD at 150 °C and an 11.6% moisture loss for the sulfadimidine/PVP ASD [59]. It has been suggested that the low hygroscopicity of Soluplus^Ⓡ^ may improve the physical stabilities of ASDs under high humidity [59].

Bilgili and coworkers investigated the effect of sodium dodecyl sulfate (SDS), along with HPC and Soluplus^Ⓡ^ on the release of griseofulvin. Soluplus^Ⓡ^ based ASDs with SDS exhibited a dramatic increase in supersaturation (max. 570%), especially at a higher Soluplus^Ⓡ^ loading, whereas no enhancement was observed for the HPC-based ASDs containing SDS. They found that griseofulvin had the better miscibility and stronger intermolecular interactions with Soluplus^Ⓡ^ than HPC. The addition of SDS increased the wettability of Soluplus^Ⓡ^-based ASDs [60]. In another study, the same research group found that the combination of amphiphilic polymers, PVPVA64 and Soluplus^Ⓡ^ can improve the wettability and solubilize the hydrophobic griseofulvin molecules through micellization [61]. Meanwhile, the Soluplus^Ⓡ^ based ternary ASD exhibited the synergistic rapid drug release and the prolonged supersaturation [61].

### 2.2 Cellulosic polymers

Cellulosic polymers are polymers derived from naturally occurring celluloses, which are the most abundant biopolymers in the world [14,15]. Cellulose is the chief structural component of plants and exhibits a fascinating structure and many interesting properties. The cellulose has low solubility in water. Hence, it is chemically modified to cellulose esters or ether derivatives, such as HPC, HPMC, HPMCP, HPMCAS, etc., which are water-soluble or moderately water-soluble [14,15]. Cellulose derivatives are widely used excipients for coatings, film, and emulsion formulations [26]. To date, there have been a number of ASD products based on this type of polymer that were approved by the FDA, as shown in Table 1.

#### 2.2.1 Hydroxypropyl cellulose (HPC)

HPC is a nonionic cellulose derivative with excellent thermoplastic properties and a low melt viscosity. It is a semisynthetic cellulose ether and is available with various chain lengths [62]. HPC is an excellent polymeric matrix for the HME process. HPC is also applied in many applications, such as extruded films, due to its good film-forming property and high mechanical strength [14]. Low-substituted HPCs (L-HPCs), such as Klucel EF or LF, are common polymeric carriers used in ASD formulations [63,64]. Samsca^Ⓡ^ is a commercial ASD product formulated with HPC and the poorly soluble drug tolvaptan [6].

Garcia et al. reported that mebendazole (MBZ) ASDs with different L-HPC contents exhibited remarkable increases in dissolution rates relative to those of crystalline drugs [63]. A pharmacodynamics study showed that the anthelmintic effects of these ASDs were significantly enhanced compared to crystalline MBZ [63]. Bachmaier et al. studied the effects of addition of low-viscosity HPC on HPMC-based ternary itraconazole ASD. The results indicated that the oral bioavailability of HPC—HPMC based ternary ASD was the highest. This result was well correlated with the *in vitro* studies of the biphasic supersaturation assays, which revealed that the addition of low-viscosity HPC increased content of drug into the organic solvent layer [62].

#### 2.2.2 Hydroxypropyl methyl cellulose (HPMC)

HPMC is a nonionic hydrophilic cellulosic derivative frequently used in manufacturing ASDs. The marketed ASDs based on HPMC are shown in Table 1. HPMC does not show a pronounced difference between the *T*_g_ and the degradation temperature [65]. In addition, HPMC has a high melt viscosity, which is not suitable for manufacturing ASDs via HME [15]. Thus, HPMC-based ASDs are mainly produced by solvent evaporation or spray drying [66], [67], [68]. Recently, a new grade of HPMC with a polymer substitution architecture, AFFINISOL™ HPMC HME (from Dow Chemical Co.), was designed to enable thermal processing during melt extrusion. The AFFINISOL™ HPMC HME shows a significantly lower melt viscosity compared to other grades [69]. Gupta et al. studied the thermal and viscoelastic properties of the AFFINISOL™ HPMC HME and showed that it can be extruded over a wider temperature processing window than PVP/VA [24].

Since HPMC contains hydrophilic and lipophilic groups, it shows excellent miscibility with a diversity of drugs [16]. Chavan et al. reported that the ASDs of nifedipine and HPMC (with drug loadings below 70%) were stable under accelerated stability conditions due to miscibility between the drug and HPMC [70]. In a study of the ASD comprising tacrolimus and HPMC, the C=O and O–H groups of tacrolimus were shown to interact with the functional groups of HPMC at the molecular level [71]. *In vivo* pharmacokinetic experiment with dogs showed that the AUC of tacrolimus in the HPMC-based ASD was significantly enhanced in comparison with the crystalline drug [71].

HPMC was used along with other polymers to enhance the performance of ASDs [66,^68^,72]. For instance, Lee et al. prepared celecoxib ASDs containing two polymers, PVP and HPMC, by spray drying [72]. The results showed that the dissolution rates of the ASDs containing both PVP and HPMC were much higher than those of ASDs containing PVP or HPMC alone [72].

#### 2.2.3 Hypromellose acetate succinate (HPMCAS)

**Fig. 3:**
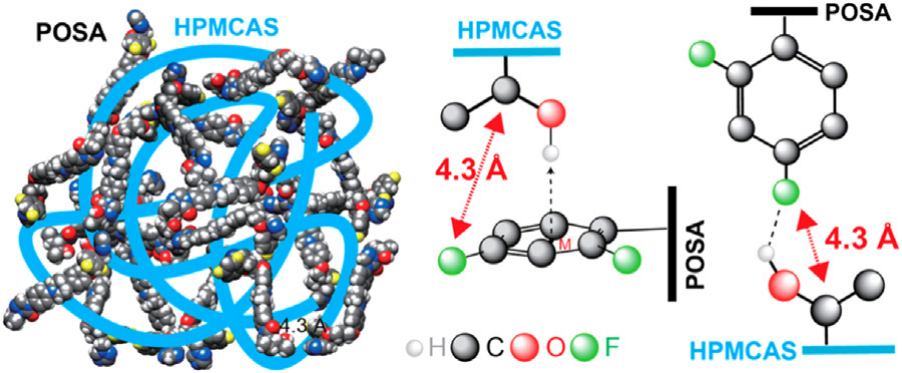
Two hydrogen-bonded patterns formed between POSA and HPMCAS . Adapted from [78] with the permission.

HPMCAS is modified from HPMC by esterification with acetic acid anhydride and succinic acid anhydride [26]. HPMCAS was first introduced as an enteric coating material with the three grades L, M, or H according to the contents of the acetyl substituents [26]. HPMCAS has been used extensively in formulating ASDs, and it shows significantly enhanced solubility, physical stability and manufacturing reproducibility [14,15]. HPMCAS has a relatively low *T*_g_ and a high degradation temperature, so it is suitable for manufacturing ASDs via the HME method [73]. Compared to other polymers used in ASDs, HPMCAS has many advantages, such as lower hygroscopicity, stronger intermolecular interaction of drug and polymer, and greater drug supersaturation during dissolution [74]. HPMCAS may form strong ionic interactions with weakly basic drugs due to it containing acid group [75], [76], [77], [78]. Lu et al. performed ^1^H−^19^F correlation experiments to probe the drug-polymer interactions between posaconazole (POSA) and HPMCAS in an ASD [78]. Solid-state NMR experiments revealed interactions between the hydroxyl groups of HPMCAS and the difluorophenyl groups of POSA [78]. Two types of hydrogen bond (O−H···F and O−H···Ph) were evidenced based on the measured ^13^C−^19^F distances (Fig. 3) [78].

The number of acetate substituents on HPMCAS could impact the extent of drug-polymer interactions in ASD. Ishizuka et al. applied FTIR and solid-state ^13^C CP/MAS NMR to investigate the molecular interactions between carbamazepine (CBZ) and HPMCAS with different substituent contents [79]. They found that compared to HPMCAS-HF, recrystallization of amorphous CBZ was more effectively inhibited by HPMCAS-LF due to its higher percentage of succinate groups [79].

HPMCAS is a weakly hygroscopic polymer; thus, under high humidity, the ASD containing HPMCAS has better physical stability than ASDs containing hygroscopic polymers [16]. Konno et al. evaluated the effects of HPMC, HPMCAS and PVP on the felodipine crystallization. Compared to PVP, HPMC and HPMCAS were superior in resisting crystallization after exposure to moisture [80]. The ASD containing felodipine and HPMCAS exhibited less moisture absorption [80].

HPMCAS is highly effective in inhibiting drug crystallization from supersaturated solutions [81], [82], [83], [84]. Xie et al. investigated the impacts of polymer types on the crystallization and dissolution rates of celecoxib ASDs [81]. HPMCAS was more effective in maintaining supersaturation than PAA [81]. In another study, the effectiveness of polymers in maintaining supersaturation was investigated with supersaturated solutions containing different predissolved polymers. HPMCAS maintained the supersaturated solution of celecoxib for more than 8 h, and crystallization of the supersaturated celecoxib was observed within 60 min for PVP [82].

The HPMCAS grades also influence dissolution rates of ASDs. Repka and coworkers studied the HPMCAS grade required for generation of stable ASDs with efavirenz and nifedipine. The dissolution results for nonsink conditions (pH 6.8) showed that only the L grade solubilized efavirenz, while the M and H grades were similar to efavirenz API [85]. On the other hand, nifedipine was solubilized by all three polymers, but L grade formulations had a higher initial release [85]. The HPMCAS L grade is more ionized in pH 6.8 because it has the highest ratio of succinoyl groups among all grades, leading to differences in the dissolution rates [85].

The pH affected HPMCAS aggregation in solution, which may influence its ability to maintain supersaturation of the ASDs. Qian and coworkers found that the HPMCAS aggregation and drug/HPMCAS affinity increased with decreasing pH, and the supersaturation of posaconazole in a HPMCAS solution was pH dependent [86]. They concluded that HPMCAS aggregation and the drug/HPMCAS affinity were the key factors governing the duration of supersaturation [86]. Bristol et al. also found that the conformation of HPMCAS in solution played an important role in maintaining supersaturation of ASDs [87]. The changes in conformations from random coil to aggregation significantly increased celecoxib supersaturation [87].

#### 2.2.4 Hydroxypropyl methylcellulose phthalate (HPMCP)

HPMCP is modified from HPMC and is a phthalic half ester [14,15]. There are two types of HPMCPs in the market with different solubilities (HP-55 and HP-50) [14,15]. HPMCP is often used as an enteric coating material to prevent drug degradation in gastric acid [14,15]. Based on the chemical structure of HPMCP, the phthalyl acid group is expected to be a key functional group in bonding with drugs, and HPMCP has been used as the matrix with which to form ASDs in some studies [88], [89], [90], [91], [92]. Nie et al. reported that the ASD of clofazimine and HPMCP exhibited superior physical stability even with more than 60% drug loading [90]. The authors proposed that protonated clofazimine was bound to the carboxylate functional groups of HPMCP to form ion pairs based on spectroscopic characterization and quantum chemistry calculations [90]. Solid-state ^13^C NMR spectroscopy confirmed the presence of ionic interactions between clofazimine and the carboxylates in the phthalyl acid substituents of HPMCP in the ASD [91]. In a study of lapatinib-HPMCP ASDs, ^15^N ssNMR showed strong ionic interactions between the drug and HPMCP, which was also indicated by the positive difference in the *T*_g_ value of the ASD compared to the value predicted with the Couchman−Karasz equation [92].

#### 2.2.5 Cellulose acetate phthalate (CAP)

CAP is derived from phthalic anhydride and is a partial acetate ester of cellulose containing acetyl and phthalyl groups [26]. DiNunzio et al.studied effects of CAP on the bioavailabilities of itraconazole ASDs [93]. The results showed that the ASDs containing CAP shows greatest degree of supersaturation [93]. The bioavailability of *in vivo* testing in a rat showed a 2-fold improvement compared to that of the marketed product [93]. There have been few reports of ASDs formulated with CAP due to the limited miscibility between drug molecules and CAP [16].

### 2.3 Acrylate and methacrylate (co-) polymers

Eudragit^Ⓡ^ is a methacrylic acid copolymer with various structures and characteristics depending on its substituents [26]. Eudragit^Ⓡ^ has been widely used in forming enteric film coatings, taste/smell masking, sustained release, and moisture prevention. These polymers are also used as carriers in manufacturing ASDs [94], [95], [96], [97].

#### 2.3.1 Eudragit^Ⓡ^ E PO

**Fig. 4:**
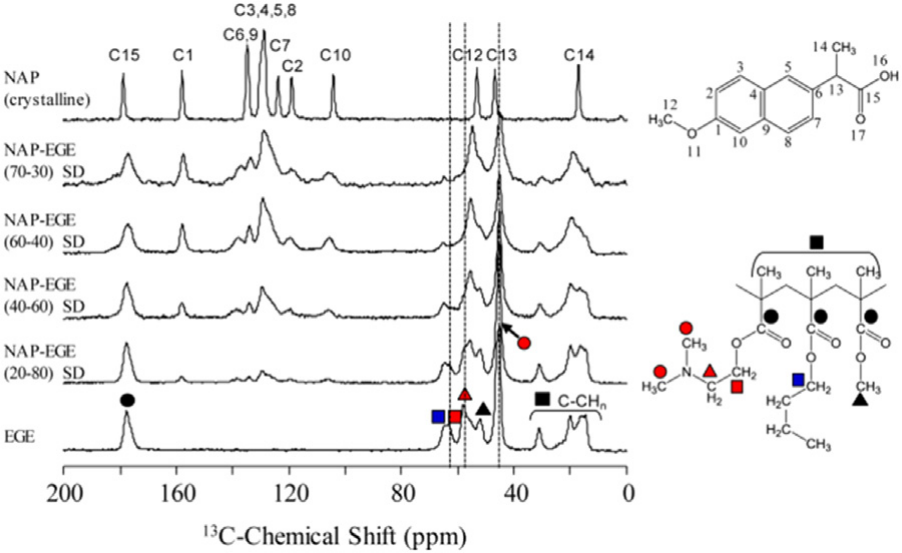
^13^C SSNMR spectra of Eudragit E (EGE), Naproxen (NAP)−EGE with different ratio ASDs, and crystalline NAP. Adapted from [99] with the permission.

Eudragit^Ⓡ^ E PO is a cationic copolymer and soluble at pH values below 5.5, while swellable at higher pHs [14,15]. Acidic drugs can form strong interactions with Eudragit^Ⓡ^ E PO [76,^94^,97]. Lubach et al. studied ASDs comprising indomethacin and Eudragit^Ⓡ^ E PO by ^15^N ssNMR, and the results showed that ionic complexes were formed between the drugs and polymers in the ASDs [98]. Sarode et al. studied indomethacin-Eudragit^Ⓡ^ E PO interactions with FT-IR spectroscopy and concluded that this specific interaction was improved upon storage at elevated temperature and humidity [76]. Ueda et al. found that the amino groups of Eudragit^Ⓡ^ E PO (EGE) were important for the ionic interactions formation with the carboxylic groups of Naproxen (NAP), as evidenced by peak shifts observed in the ^13^C SSNMR spectra (Fig. 4) [99].

Frank et al. investigated the release mechanisms of the weakly basic pharmaceuticals posaconazole and lumefantrine ASDs containing Eudragit^Ⓡ^E PO, HPMCAS or PVP/VA. Their results showed that the basic Eudragit^Ⓡ^ E PO enabled rapid drug release at low pH due to its high solubility in acidic solutions [100]. This study provided a new way to increase bioavailability of acidic drugs by using basic polymers as stabilizers in ASDs [100]. The ASDs prepared with Eudragit^Ⓡ^ E PO also generates stable supersaturated solutions with high drug concentrations [94,^97^,101]. The ASD of curcumin and Eudragit^Ⓡ^ E PO formed highly aqueous soluble complexes through hydrogen bonding between the phenolic -OH groups of curcumin and the -C=O group of the Eudragit^Ⓡ^ E PO [101]. *In vivo* results showed that peak plasma concentrations of curcumin/Eudragit^Ⓡ^ E PO ASD were increased 6-fold and the oral bioavailability was increased by 20-fold compared to unformulated curcumin [101]. In another study, it was found that Eudragit^Ⓡ^ E PO formed micelles in acidic solutions to solubilize drugs [102]. The micelles formation increased the solubilities of ibuprofen, felodipine and bifendate by 10–100-fold [102]. Yoshida et al. also found that ASDs formed with tacrolimus and spray-dried Eudragit^Ⓡ^ E/HCl enhanced the drug solubility by forming micellar-like structures in solution [103,104].

The solubility of Eudragit^Ⓡ^ E PO is influenced by other substances in solution. Ueda et al. reported that the addition of saccharin increased the drug dissolution of a phenytoin/Eudragit^Ⓡ^ E PO ASD [105]. Solid-state ^13^C NMR and solution-state ^1^H NMR measurements indicated the presence of ionic interactions between Eudragit^Ⓡ^ E PO and saccharin, which promoted the dissolution of Eudragit^Ⓡ^ EPO and phenytoin [105]. In a further study, Okamoto et al. found that the interaction between Eudragit^Ⓡ^ E PO and saccharin increased the mobility of the Eudragit^Ⓡ^ E chains, and subsequent conversion of Eudragit^Ⓡ^ E PO to a partially folded structure over pH 4.5–6.5 resulted in enhanced drug solubility [106]. Eudragit^Ⓡ^ E PO is relatively less hygroscopic than other hydrophilic polymers; thus, it can be blended with hydrophilic polymers and reduce the negative impact of moisture on the physical stabilities of ASDs. For instance, Eudragit^Ⓡ^ E PO was blended with PVP/VA to form a ternary ASD that showed reduced moisture absorption at high temperature and humidity levels [107].

#### 2.3.2 Eudragit^Ⓡ^ L100

Eudragit^Ⓡ^ L100 is an anionic copolymer consisting of methacrylic acid and methyl methacrylate [14,^15^,26]. This polymer is ionized above pH 6.0 and can be used as enteric coating materials to stabilize ASDs [26]. Eudragit^Ⓡ^ L100 was coated onto ASDs and used as the enteric layer [108]. In the coating process, sucrose beads were first coated with a glass solution of ezetimibe, lovastatin and Soluplus^Ⓡ^ and then top-coated with an Eudragit^Ⓡ^ L100 layer, which was used to protect the drugs from the gastric environment [108]. Eudragit^Ⓡ^ L100 was also used as a carrier to form an ASD. For instance, Maniruzzaman et al. investigated ASDs comprising cationic drugs in Eudragit^Ⓡ^ L100 prepared by the HME technique [109]. Thermal analyses and molecular modeling simulations showed that Eudragit^Ⓡ^ L100 formed strong molecular interactions with amorphous drugs and achieved enhanced physical stability [109].

#### 2.3.3 Eudragit^Ⓡ^ S100

Eudragit^Ⓡ^ S100 is an anionic methacrylate copolymer that is fully ionized above pH 7.0 [26]. Eudragit^Ⓡ^ S100 enhances the poorly soluble drugs dissolution at pH values above 7.0 [110]. In a study of the ASD of celecoxib formed with the pH-sensitive polymer Eudragit^Ⓡ^ S 100, the release of the ASD at pH 7.4 was significantly higher than those of neat amorphous or crystalline drugs [110]. A good correlation was established between the *in vivo* results and the *in vitro* data obtained in a dissolution medium at pH 7.4 [110]. Eudragit^Ⓡ^ S100 forms strong intermolecular interactions that lead to enhanced physical stability in the solid-state as well as prolonged supersaturation [111].

#### 2.3.4 Eudragit^Ⓡ^ L 100–55

Eudragit^Ⓡ^ L100–55 is an anionic copolymer polymer consisting of methacrylic acid and ethyl acrylate, and it is ionized above pH 5.5 [14,^15^,26]. Eudragit^Ⓡ^ L100–55 is often used as an enteric coating agent to prevent the degradation of drugs in acidic environments [14,^15^,26]. For instance, Riekes et al. developed a solid dispersion comprising ezetimibe and lovastatin coated with a layer of Eudragit^Ⓡ^ L100–55 by the fluid bed coating technique [108]. They found that Eudragit^Ⓡ^ L100–55 slowed drug release in an acidic environment and enabled fast drug release at pH 6.8 [108]. Maniruzzaman et al. reported that the Eudragit^Ⓡ^ L100–55 ASD with propranolol HCl or diphenhydramine HCl exhibited good physical stability due to ionic interactions of drug and polymer [109]. Shah et al. prepared vemurafenib ASD by a solvent-controlled coprecipitation method and found that Eudragit^Ⓡ^ L 100–55 stabilized amorphous vemurafenib in the solid-state by inhibiting both nucleation and crystal growth [112].

### 2.4 Other polymers

#### 2.4.1 Polyvinyl acetate phthalate (PVAP)

PVAP is a vinyl acetate polymer and is soluble above pH 5. Monschke et al. prepared a PVAP-based ASD by the HME process and found that dissolution can form a stable supersaturated solution [113]. Due to the high solubility of PVAP above pH 5, the PVAP based ASD of indomethacin showed a faster dissolution rate compared with HPMCAS or Eudragit^Ⓡ^ L100–55 ASDs [113]. DiNunzio et al. produced a PVAP ASD of itraconazole by ultrarapid freezing, the *T*_g_ of which deviated from the predicted value because of hydrogen bonding interactions [93].

#### 2.4.2 Poly(acrylic acid) (PAA)

PAA is an acrylic acid polymer with pK_a_ values of approximately 4.5 [114,115]. PAA has many carboxylic groups which can form amorphous salts with basic drugs. Gui et al. produced an clofazimine (CFZ) and PAA amorphous salt via simple slurry method [114]. The amorphous salt prepared with a 75 % drug loading shows a good physical stability under accelerating conditions [114]. In contrast, the ASD containing PVP crystallized in one week under the same conditions [114]. In addition, the amorphous CFZ−PAA salt exhibited the enhanced dissolution rate, tabletability and powder flowability in comparison with the crystalline drug [114]. A similar result was also found for the ASD of lumefantrine and PAA, in which formation of the amorphous salt enhanced drug stability and release rates [116]. It has been reported that the esterification reaction involving by PAA and PVA produced crosslinked ASDs [117,118]. The crosslinked ASDs showed pronounced stability with extremely high drug loading (≥ 90%) [117,118].

#### 2.4.3 Polyethylene glycol/polyethylene oxide (PEG/PEO)

PEG is a semicrystalline polymer that is widely used in various pharmaceutical formulations [14,^15^,26]. High molecular weight PEG is usually described as PEO [14,15]. PEG/PEO shows a melting point near 60 °C, and the *T*_g_ is very low and depends on the molecular weight. The *T*_g_ rises to a maximum of −17° for a Mw of 6,000 and then decrease to −53° for PEOs with Mw higher than 200,000 [119]. Poorly water-soluble drugs dispersed within semicrystalline polymers have been the subject of many studies [120], [121], [122], [123], [124]. However, few marketed ASDs have utilized semicrystalline polymers, in part due to concerns about physical instability [120]. The crystallization behaviors of both PEG/PEO and the API in these ASDs will affect the microstructure and subsequently impact the dissolution process [125], [126], [127]. The tendency of the drug to crystallize and the interactions between the drug and PEG significantly affect the drug position in the PEG matrix [128]. Further investigation of the microstructures of different drug/PEG systems and the microstructural evolutions during crystallization are still needed for a better understanding of these solid dispersions [121,129]. However, due to high mobility, low-concentration PEO in ASD acted as a plasticizer and accelerated nucleation and crystal growth rates [130], [131], [132], [133].

#### 2.4.4 Poloxamer

Poloxamer is a triblock copolymer composed of poly(ethyleneoxide)−poly(propylene oxide)−poly(ethylene oxide) blocks [14,^15^,26]. Poloxamer is a semicrystalline material containing the semicrystalline PEO segments and the amorphous PPO segments. The melting point of poloxamer is around 55 °C. [14,^15^,26]. Poloxamer can be used as a polymeric carrier in ASD to enhance solubility and dissolution rate of poorly soluble drugs. Kolasinac et al. selected two grades poloxamer (poloxamer P188 and poloxamer P407) to prepare desloratadine ASDs [134]. It was found that poloxamers could significantly enhance the dissolution of the drug and the dissolution rate increased with increasing the content of poloxamer. Specifically, the ASDs containing poloxamer 188 exhibited a more rapid drug release than those containing poloxamer 407 [134]. Karekar et al. also found that poloxamer 188 showed superior performance in enhancing solubility and dissolution rate of etoricoxib [135]. Poloxamer can be often used as a surfactant in ASDs. Vasconcelos et al. investigated the presence of poloxamer 407 on the bioavailability of the resveratrol: Soluplus^Ⓡ^ (1:2) ASD. The *in vivo* results showed that AUC_o-t_ and C_max_ of the ASD containing 15% poloxamer 407 were 2.5 fold higher than the ASD in the absence of poloxamer [136].

## 3 Formulation design of ASDs

**Fig. 5:**
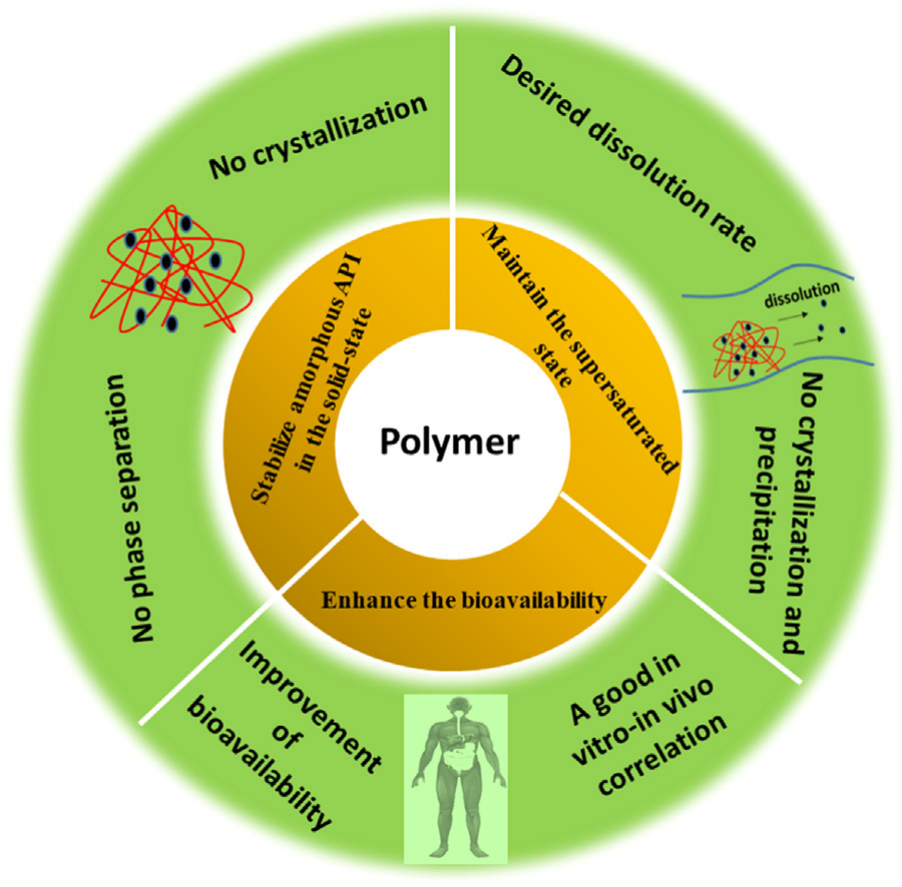
Schematic illustration of the polymer characteristics needed for ASD formation.

The selection of a suitable polymer plays an important role in performance of ASDs [2]. Ideally, a polymer in a successful ASD formulation should exhibit three characteristics: (a) it should stabilize amorphous API in the solid-state; (b) it should enhance the solubility and dissolution rate as well as maintain supersaturation in gastrointestinal conditions; and (c) it should also enhance the bioavailability by improving drug permeation through the gastrointestinal membranes [2]. A diagram showing the role of polymer in a successful ASD formulation is provided in Fig. 5.

### 3.1 Miscibility, physical and chemical stability

#### 3.1.1 Miscibility and phase separation

An appropriate polymer in ASD should effectively inhibit crystallization. Before assessing the effects of a polymeric carrier on crystallization of an ASD, the miscibility of the polymer and drug should be investigated [14,^15^,137].

The Hansen solubility parameters have been used to evaluate the miscibility of drug and polymer in order to select a suitable polymer for the ASD development [14,15]. Solubility parameters are calculated from the chemical structures of compounds using the approaches of Hoftyzer/Van Krevelen [14,15]. In this method, the dispersive forces, interactions between polar groups, and hydrogen bonding groups are taken into account:

$$\delta_{\text{coh}}^{2}=\delta_{d}^{2}+\delta_{p}^{2}+\delta_{h}^{2}$$

Where δ_d_, δ_p_, and δ_h_ are the dispersive, polar, and hydrogen bonding solubility parameter components, respectively.

For the evaluation of miscibility, it has been suggested that if the difference between the solubility parameters of the components to be mixed (δ) is smaller than 7 MPa^1/2^, then the components are likely to be miscible, and if δ is smaller than 2 MPa^1/2^, the components might form a solid solution. δ values larger than 10 MPa^1/2^ suggest the immiscibility between the components. This method is useful for the polymer selection of ASDs [138,139], but sometimes the results obtained by this method are inconsistent with the method of melting point depression [140].

The miscibility of multiple components in an ASD formulation is governed by free energy of mixing: Δ*G*_mix_ = Δ*H*_mix_ − *T*Δ*S*_mix_. Mixing occurs if Δ*G*_mix_ < 0. Flory-Huggins (FH) theory is a more widely applied approach to estimate drug–polymer miscibility [14,^15^,137]. The Flory-Huggins interaction parameter χ can be calculated via the following steps.

First, the solubility of drug in a polymer is used to calculate drug's activity *a*_1_:

$$\text{ln}\alpha_{1}=\left(\Delta H_{m}/R\right)\left(1/T_{m}-1/T\right)$$

where *T*_m_ is the melting point, Δ*H*_m_ is molar heat of melting, and *T* is the experiment temperature.

Then, the activity of drug in polymer is given by

$$\text{ln}\alpha_{1}=\;\text{ln}\upsilon_{1}+\left(1-\frac{1}{x}\right)\upsilon_{2}+\chi \upsilon_{2}^{2}$$

where *v*_1_ and *v*_2_ are the volume fraction of drug and polymer, *x* is molar volume of the polymer /drug, and χ is interaction parameter. A negative value of χ suggests that drug is thermodynamically miscible with polymer.

**Fig. 6:**
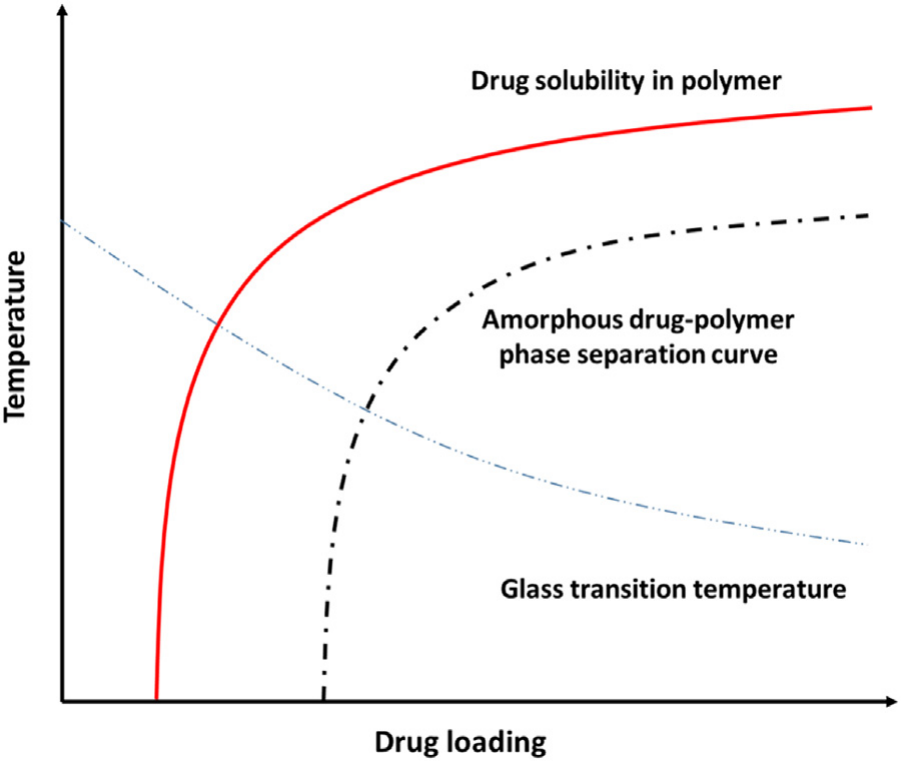
A typical temperature-composition phase diagram for an ASD.

A phase diagram can also be constructed during χ determination, and a simplified drug–polymer phase diagram is shown in Fig. 6 [141], [142], [143], [144]. The solubility curve forms the boundary between the thermodynamically stable and unstable regions [49,^141^,^145^,146]. The solubility curve is particularly important in selecting a suitable polymer and determines the maximum drug loading in the ASD [49,^52^,[147], [148], [149], [150], [151]. In the ideal ASD formulation, drug dissolves into polymer and forms a molecular dispersion that is thermodynamically stable at its storage temperature.

Phase separation by ASDs poses a significant challenge for physical stability, and it promotes crystal formation and causes the ASD to shift to a lower energy state [142,152]. As shown in Fig. 6, below the phase separation curve, amorphous phase separation takes place spontaneously, and within position between phase separation curve and solubility curve, local fluctuations of drug and polymer contents might be required to cause destabilization [142]. It is necessary to investigate the potential phase separations and inhomogeneities in ASD. Qian et al. found that a single *T*_g_ may not always be a reliable phenomenon of the homogeneity of an ASD [153]. By taking advantage of confocal Raman mapping, the localized compositional distributions of ASDs were found to be better correlated with the physical stabilities [153]. ssNMR can be used to predict phase separations of ASDs with sub-100 nm resolution [154], [155], [156]. In a study of spray-dried ASDs composed of AMG 517 and HPMCAS, ssNMR showed that the drugs and polymers were in intimate contact over a 10–20 nm range [156]. Yuan et al. investigated nifedipine/PVP ASD phase separations at drug loadings greater than 90% with ssNMR relaxometry and found that the phase separation domains were greater than *Ca*. 20 nm in size [157]. Taylor and coworkers investigated the miscibilities of amorphous telaprevir with three polymer excipients: HPMC, HPMCAS, and PVP/VA [158]. The domain sizes of the drug-rich regions on the ASD surfaces varied from 50 nm to 500 nm, as shown by atomic force microscopy coupled with infrared spectroscopy (AFMIR) [158]. Three polymers showed different miscibilities with the drug, and the phase separations of ASDs containing HPMC and PVPVA was found when drug content is higher than 10%, and phase separation occurred at drug loadings above 30% in the presence of HPMCAS [158]. The hygroscopicity of a polymer also impacts phase separation during the storage of an ASD. For example, phase separation was observed for PVP-based amorphous celecoxib, while HPMCAS-based ASDs did not show any phase separation [159]. The water in an ASD can act as a plasticizer to destabilize the ASD [47,160]. Kapourani et al. constructed moisture-induced thermodynamic phase diagrams for rivaroxaban ASDs with different polymeric carriers, and this showed that PVP- and PVP/VA-based ASDs were easier to phase separation under elevated RHs compared to Soluplus^Ⓡ^ and HPMCAS systems [41].

#### 3.1.2 Crystallization

In reality, the drugs loaded in ASDs are often present at levels above the drug solubility in polymer. In this case, a kinetically stable ASD formulation can be prepared by selecting the appropriate polymer carrier, polymer/drug ratio and manufacturing parameters. The impacts of polymers on the crystallization kinetics of ASDs have been well studied [8], [9], [10], [11], [12], [13]. Several mechanisms have been pointed out to interpret the effects of polymers on crystallization [8], [9], [10], [11], [12], [13].

##### 3.1.2.1 Antiplasticization and segmental mobility of the polymer

A polymeric additive can decrease the molecular mobility of drugs in ASD due to its high *T*_g_ termed “anti-plasticizing effect”, which leads to decreasing crystallization rates [10]. Recently, Yu and coworkers reported that 1% polymers strongly altered the drugs crystallization in the glassy state [8,9]. There was a strong correlation between the *T*_g_ of polymer and inhibiting effects [8,9]. A master curve has been used to describe the effects of polymers on the crystallization of amorphous drugs by (*T_g_*,_polymer_ − *T*_g_,_drug_)/*T_cryst_* [9].

##### 3.1.2.2 Polymer-drug interactions

**Fig. 7:**
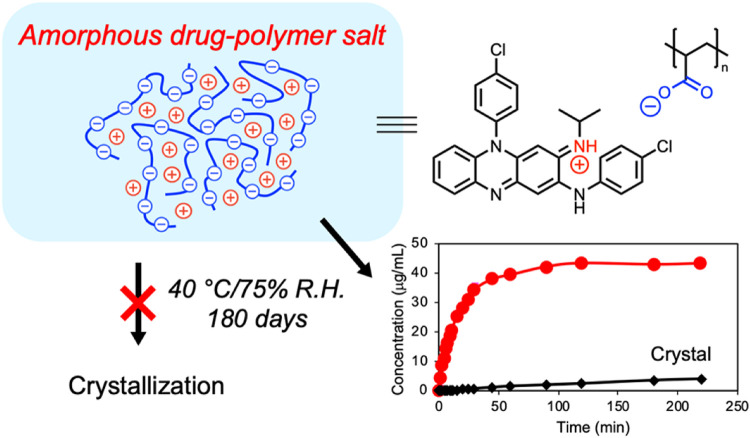
A schematic model for the ionic interactions between clofazimine and PAA. Adapted from [114]with the permission.

Polymer-drug interactions such as ionic, hydrogen bonding and van der Waals interactions are known to govern the physical stabilities of ASDs [14,^15^,^32^,[161], [162], [163], [164]. For instance, Taylor and coworkers evaluated the impacts of chemically different polymeric additives on the crystallization of two poorly water-soluble drugs (bifonazole and nimesulide) [161]. They found that hydrogen bonding was a key factor in preventing crystallization of amorphous drugs in ASDs [161]. Kothari et al. evaluated the impacts of hydrogen bonds on the physical stabilities of nifedipine ASDs made with three different polymers [32]. The strengths of the drug-polymer hydrogen bonds, the structural relaxation times and the physical stabilities all decreased following order PVP > HPMCAS > PAA [32]. The strongest drug-polymer interaction in PVP-based ASD provided the greatest inhibition of amorphous drug crystallization [32]. It has been reported that strong ionic interactions in ASDs can significantly enhance the physical stabilities of ASDs. Yu and coworkers reported several cases in which amorphous drug–polymer salts were formed and detailed the pharmaceutical benefits of using the acidic polymer PAA [114,116]. For instance, clofazimine formed strong ionic interactions with PAA, and the clofazimine/PAA ASD was remarkably stable against crystallization (as shown in Fig. 7) [114]. Similar results were observed for the ASD of lumefantrine and PAA [116]. In contrast, the ASD containing un-ionized lumefantrine in PVP crystallized completely within 4 days [116].

##### 3.1.2.3 Molecular mobility

**Fig. 8:**
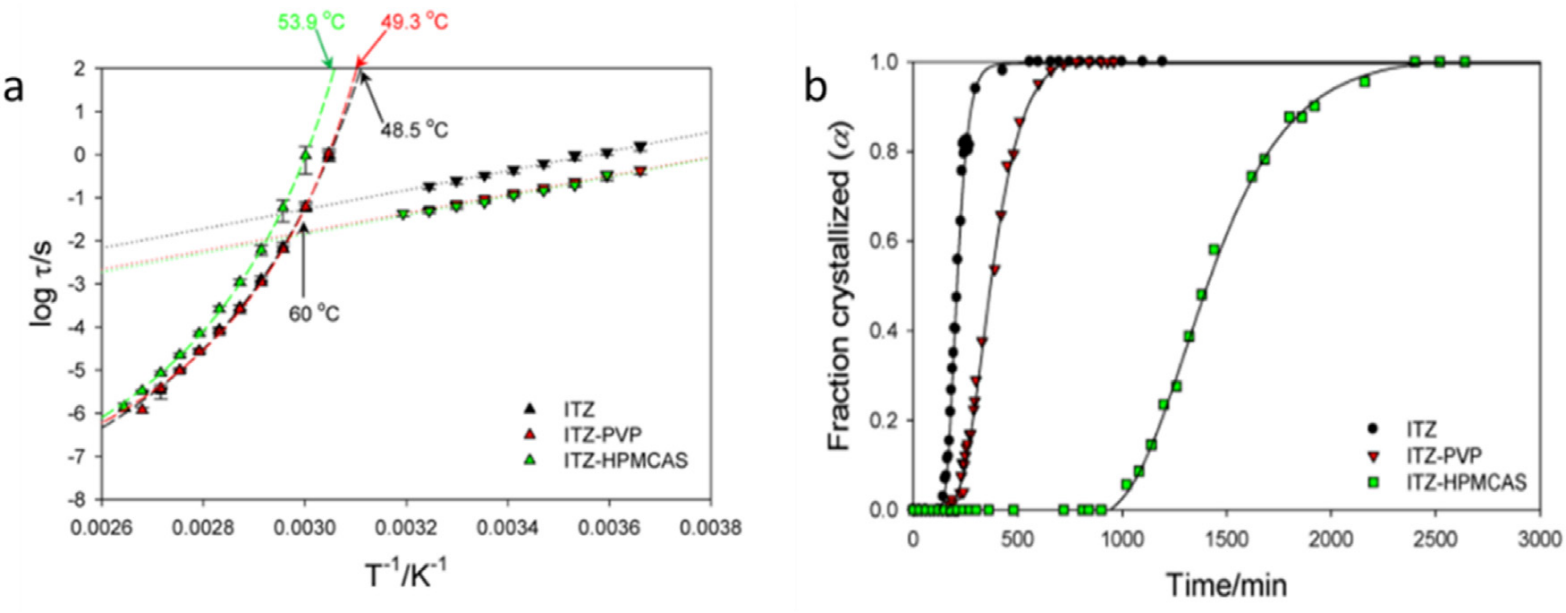
The α-relaxation and β-relaxation times for different systems *vs* temperature (a). Crystallization for different systems *vs* time at 82 °C (b). Adapted from [77] with the permission.

Molecular mobility is also one of the most important factors governing crystallization of ASDs [77,^157^,[165], [166], [167]. Dielectric spectroscopy has been widely applied to characterize the molecular mobilities of amorphous drugs [77,168]. Paluch and coworkers used broadband dielectric spectroscopy and investigated the molecular mobility and crystallization of pure nimesulide and the ASDs formed with inulin, Soluplus^Ⓡ^, and PVP respectively [168]. The molecular mobilities of the pure drug and the binary drug-polymer mixtures were characterized by broadband dielectric spectroscopy [168]. The greatest inhibition of crystallization and reduction in molecular mobility were observed for the ASD containing PVP. The authors suspected that the antiplasticization effect and steric hindrance from the polymer affected the molecular mobility and inhibited crystallization [168]. Suryanarayanan and coworkers characterized the molecular mobilities of itraconazole (ITZ) in ASDs containing PVP or HPMCAS by using broadband dielectric spectroscopy [77]. The dielectric spectra (Fig. 8a) showed that the α-relaxation times of ITZ were substantially increased in the presence of HPMCAS, while PVP had a negligible effect on α-relaxation times. An isothermal crystallization study showed that HPMCAS was more effective in inhibiting crystallization compared to PVP (Fig. 8b), suggesting a good correlation between α-relaxation and crystallization kinetics [77]. Kothari et al. compared the molecular mobilities of amorphous nifedipine ASDs formed with PVP, PAA, and HPMCAS [32]. Dielectric spectroscopy showed that addition of the three polymers enhanced the physical stabilities by reducing molecular mobility [32]. The strengths of the drug-polymer hydrogen bonds, the structural relaxation times, and the physical stabilities all decreased following order PVP > HPMCAS > PAA, suggesting that drug-polymer interactions affect the molecular mobility in ASD systems [32].

A polymer with a low *T*_g_ and high segmental mobility can increase the molecular mobility of an amorphous drug and decrease the physical stability via the “plasticization” effect [132,133]. It was reported that low-concentration PEO significantly enhanced the crystallization of amorphous griseofulvin. The liquid dynamics of amorphous griseofulvin with and without PEO were characterized by dielectric spectroscopy. The α-relaxation times of drugs decreased with increasing PEO content, thus increased in global molecular mobility [132].

Ueda et al. investigated the abilities of Eudragit^Ⓡ^ L, HPMCAS, and PVP/VA to decrease amorphous felodipine crystallization [169]. The molecular mobilities of the drugs in the ASDs were evaluated with solid-state ^13^C spin-lattice relaxation time measurements [169]. They found that the molecular mobility of drug was strongly inhibited by the polymers at the storage temperature, and mobility decreased in the order PVP/VA > HPMCAS > Eudragit^Ⓡ^ L [169]. Good correlations between drug mobility and physical stability were observed for those ASD systems [169]. Sahoo et al. evaluated the impacts of crosslinking on the molecular mobilities and physical stabilities of KTZ ASDs [117]. PAA and PVA were selected as polymer and crosslinker, respectively [117]. Sahoo et al. found that the molecular mobility was progressively reduced with increasing in the crosslinker content, and physical stability was attendantly increased [117].

##### 3.1.2.4 Nucleation

The crystallization of drug molecules from ASDs typically consists of nucleation and crystal growth processes. More attention has been focused on the effects of additives on crystal growth [9], [10], [11], [12], [13]. However, nucleation is also the critical factor affecting the physical stability of ASDs [170]. Since the nucleation process is still poorly understood, thus the impacts of polymers on nucleation of amorphous drugs are debatable [11,[171], [172], [173], [174], [175], [176]. Taylor and coworkers found that nucleation rates of amorphous acetaminophen were increased by the strong hydrogen bond donors in PAA, but HPMCAS, which contains weaker H-bond donor and acceptor, exhibited the strongest inhibition of nucleation. This study showed that there is no direct correlation between nucleation rate and any easily identifiable system property, including drug-polymer interactions, *T*_g_ [172].

**Fig. 9:**
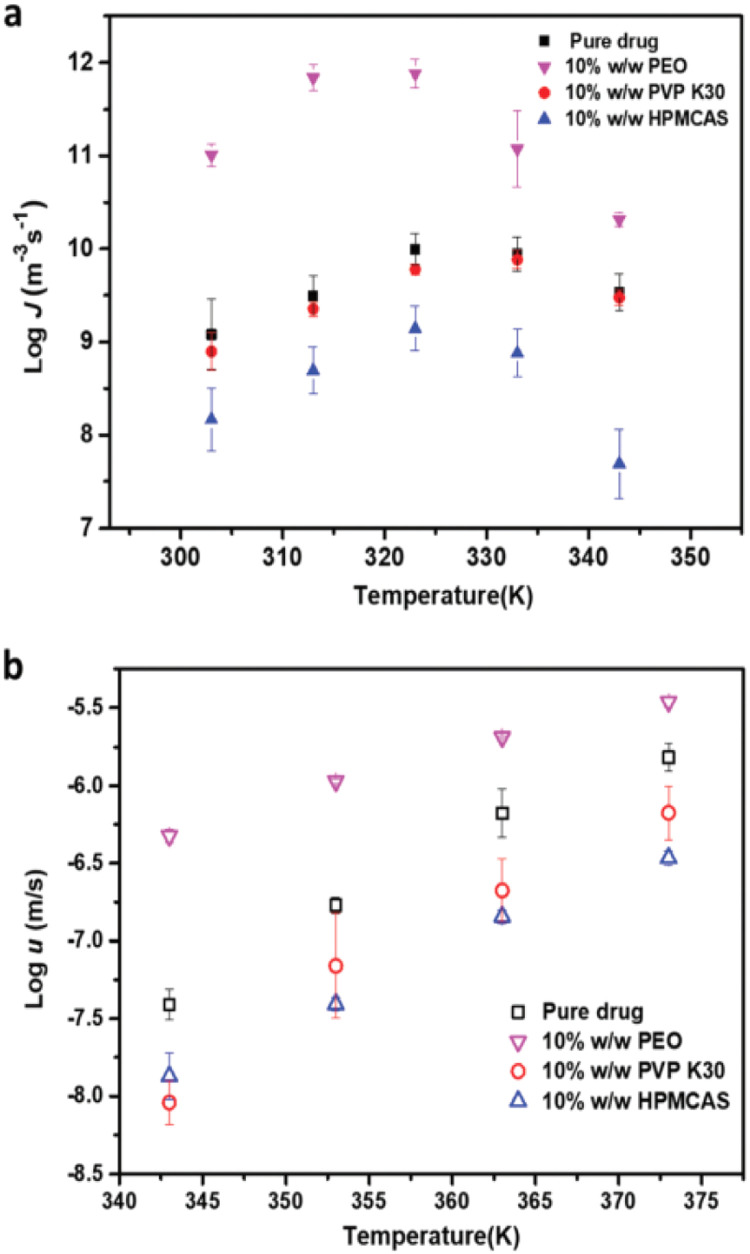
The impacts of different polymers on the nucleation rate (*J*) and growth rate (*u*) of fluconazole Form II. Adapted from [131] with the permission.

Recently, Yu and coworkers reported that the PVP has the same inhibitory effects on nucleation and crystal growth of D-sorbitol and D-arabitol [176]. This suggested that the two processes may have similar kinetic barriers and that polymers slow the two processes in a similar way by influencing the molecular motions [176]. Zhang et al. compared the crystal nucleation and growth rates of fluconazole with different polymeric additives, including HPMCAS, PVP, or PEO (10%, w/w) [131]. The HPMCAS had the largest inhibitory impact on the nucleation of fluconazole. In contrast, the PEO significantly increased nucleation (Fig. 9) [131]. Consistent with the earlier results, these polymers influence two processes of fluconazole to a similar extent, indicating that the two processes have a similar kinetic barrier [131].

#### 3.1.3 Chemical stability

Drug degradation may occur during the manufacturing of ASDs. Polymers may influence the degradation behaviors and chemical stabilities of ASDs [177,178]. Alvarenga et al. studied the extent of ritonavir degradation in ASDs formulated with PVP, PVP/VA, HPMCAS, and HPMC during isothermal heating and HME. The HPMCAS and HPMC accelerated drugs degradation, while PVP and PVP/VA reduced degradation rates. The authors found that molecular mobilities and intermolecular interactions influenced the degradation temperatures of the ASDs [177]. Moseson et al. also found that polymers slowed the degradation of posaconazole compared to pure drug, and PVP/VA prevented posaconazole degradation more effectively than HPMCAS [178]. Saraf et al. studied the oxidative degradation of nifedipine in ASDs prepared with PVP of different molecular weights [179]. The PVP K30-based ASD exhibited a higher oxidative degradation rate than the ASD formulated with PVPK90 [179].

### 3.2 Dissolution and maintenance of supersaturated solutions

**Fig. 10:**
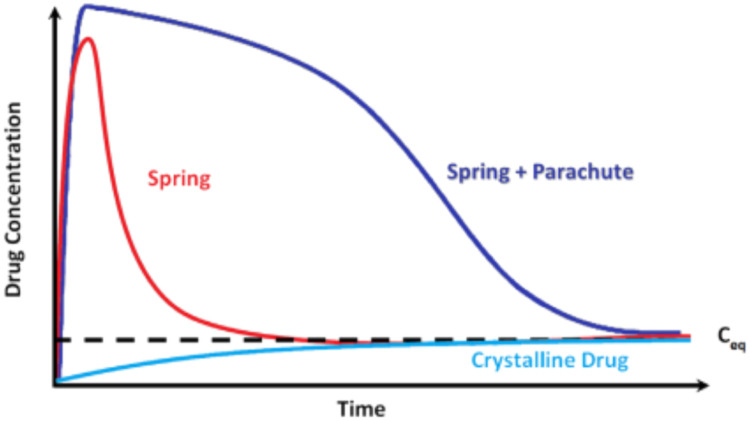
Graphical illustration of the spring and parachute effect. Adapted from [181] with the permission.

For an ASD, dissolution and supersaturation are analogous to the spring and parachute. The spring is typically a high-energy amorphous form of the drug that facilitates dissolution and supersaturation. The parachute is a process that delays crystallization of drug and maintains a supersaturated solution [180]. Fig. 10 shows a graphical illustration of the spring and parachute effects [180]. An ASD formulation showing the spring and parachute profile during *in vitro* dissolution testing holds promise for optimizing *in vivo* bioavailability [180]. The polymer in the ASD, if selected appropriately, prevents the drug against solution-mediated crystallization and maintains the supersaturated state in aqueous environments.

#### 3.2.1 Dissolution

Dissolution is often studied during the formulation development process to predict the *in vivo* behavior of the formulation. The dissolution rate is interpreted with the Noyes-Whitney relationship given by dC/dt=DA/h(Cs-Ct), where *Cs* is the saturation solubility, *Ct* is the concentration in the solution, *A* is the surface area of the drug particles, *h* is the thickness of the boundary layer separating the bulk media, and *D* is the diffusion coefficient of the drug [182]. The ASD has an obvious advantage in its dissolution rate relative to that of a crystalline drug. ASDs can increase dissolution rates by (a) increasing the apparent solubility, (b) increasing *A* by reducing the drug particle sizes, and (c) enhancing the drug wettability [14,15].

##### 3.2.1.1 Physical stabilities of ASDs

**Fig. 11:**
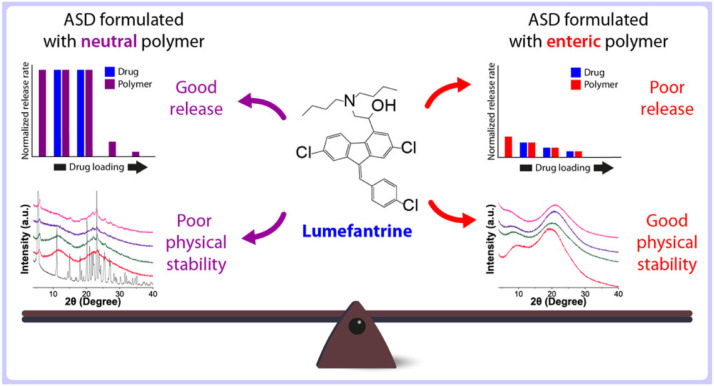
Schematic representation of the impacts of polymers on balancing solid-state stabilities and dissolution rates of lumefantrine ASDs . Adapted from [184] with the permission.

Purohit et al. developed a generic tacrolimus amorphous formulation, and crystallization of the tacrolimus ASD was intentionally induced through exposure to moderate temperatures and high relative humidity [183]. An *in vivo* study showed that the partially or fully crystallized tacrolimus ASD exhibited lower *AUC* and *C*_max_ compared to the freshly prepared ASD [183]. A polymer in an ASD may have different effects on the physical stability and the drug release [184]. Therefore, a balance between physical stability and dissolution rate should be taken into account when selecting polymers for formulation development. Hiew et al. compared the solid-state stabilities and release rates of ASDs containing a weakly basic drug, lumefantrine [184]. When formulated with a neutral polymer, the PVP/VA based ASD exhibited fast release but rapid crystallization during storage, while the enteric polymers significantly inhibited crystallization of ASDs but had poor drug release (Fig. 11) [184].

##### 3.2.1.2 Polymer content

**Fig. 12:**
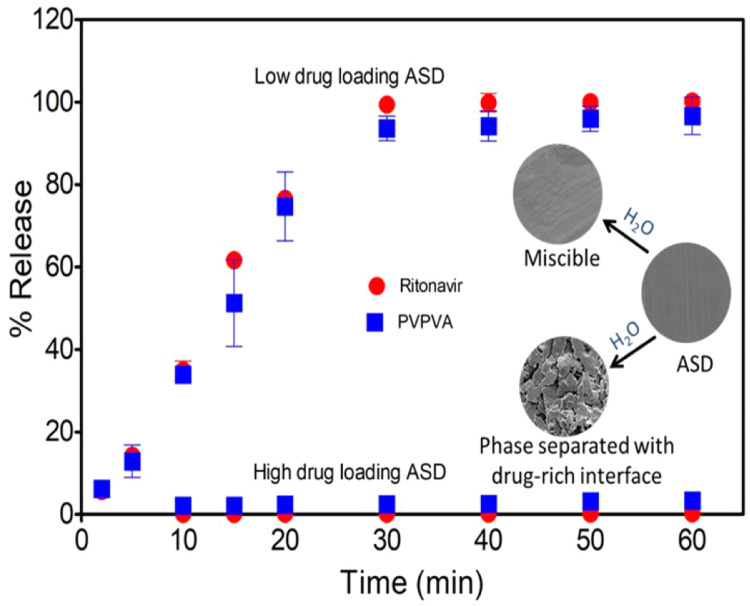
Percentages of RTV and PVPVA release from ASDs with different drug loadings . Adapted from [185] with the permission.

It is worth noting that the polymer content influences drug release from the ASD [185]. Indulkar et al. evaluated the dissolution performance of ASDs with ritonavir (RTV) and PVP/VA [185]. They found that low drug loadings (DLs) ASDs exhibited rapid, complete, and congruent release of the drug and polymer [185]. ASDs with high DLs showed slow release of both the drug and polymer and dissolution rates weresimilar to that of the pure drug (Fig. 12) [185]. In the further study, they found that ASD tablet surface partially dissolved, and high DLs gave porous surfaces and water-induced phase separation, whereas low DLs retained the miscibility [185]. These results suggested that ASD dissolution were governed primarily by the polymers at low DLs, whereas the amorphous drugs controlled dissolution at high DLs [185]. Similar behavior was identified during the dissolution of ASDs containing nilvadipine (logP = 3.04) and cilnidipine (logP = 5.54) [186]. Rapid, congruent release of the drug and the PVP/VA occurred at low DLs (< 20%, w/w), while at higher DLs, incongruent release due to slow release of the drug was observed [186]. The DL where the transition from congruent to incongruent release begins has been defined as the limit of congruency (LoC). Below the LoC, drug and polymer percent release rates are similar, and in this congruent release regime, the drug and polymer release to completion. While above the LoC, the drug and polymer release become incongruent. At even higher drug loadings, both polymer and drug release are inhibited. According to the release rate, the drug loading can be determined by LoC [187].

##### 3.2.1.3 Hydrophilicity of the polymer

The dissolution behaviors of ASDs can depend on both the drugs and polymers [188]. The water solubility of the polymer can influence drug release from the ASD [14,^15^,189]. The solubilities of some common polymeric carriers used in ASDs depend on the pH of the solution. PVP, PVP/VA, Soluplus^Ⓡ^, HPC, and HPMC are soluble under all pH conditions, while HPMCAS, HPMCP, and Eudragit^Ⓡ^ L are insoluble under acidic conditions (pH < 5.5) [14,15]. Eudragit^Ⓡ^ E PO is soluble below pH 5 [14,15]. Li et al. investigated the dissolution behaviors of quercetin ASDs made with different polymers [189]. Drug release from HPMCAS-based ASDs was quite slow and incomplete because of low hydrophilicity, while drug release from PVP-based ASDs was much faster and more complete due to the higher hydrophilicity [189].

**Fig. 13:**
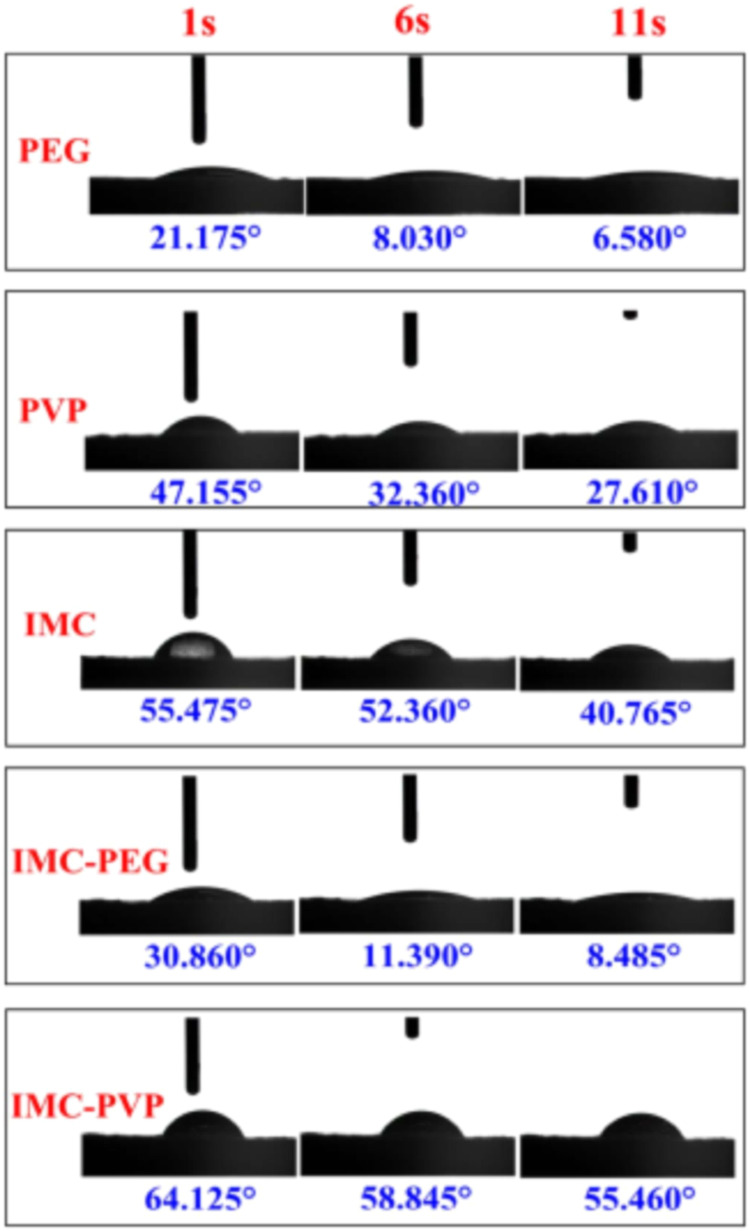
The contact angles of water on IMC, IMC-ASD tablets. Adapted from [190] with the permission.

The contact angles can be used to determine the hydrophilicities of polymers when evaluating the wetting behaviors of ASDs [190], [191], [192]. Li et al. conducted water contact angle measurements on tablets of indometacin (IMC) ASDs and their individual components. Fig. 13 shows that the water contact angle on IMC-PEG was lower than that on IMC alone, while that on IMC-PVP was higher [190]. The contact angle studies were well correlated with the *in vitro* dissolution data, where IMC-PEG showed enhanced dissolution [190].

##### 3.2.1.4 Phase separation and crystallization during dissolution

During the dissolution of ASDs, it is of very importance to maintain amorphous materials without phase separation and crystallization [14,^15^,^187^,^193^,194]. The solution-mediated phase separations and crystallizations of ASDs can be affected by drug loading, polymer type, and strengths of intermolecular interactions [195]. Polarized light microscopy (PLM), fluorescence microscopy or Raman spectroscopy have been applied to investigate the phase separation and crystallization characteristics of ASDs [82,^195^,196]. Saboo et al. investigated the phase separation of nilvadipine and cilnidipine ASDs during dissolution [186]. They found that at higher DLs, the phase separation of the ASD occurred faster compared to low DLs, and the phase separation was confirmed by energy-dispersive X-ray (EDX) and X-ray photoelectron spectroscopy (XPS) [186]. Xie et al. used PLM to investigate the phase behavior of the celecoxib-PAA and celecoxib-HPMCAS ASDs during dissolution. A rapid dissolution of the solid matrix, upon contact with the dissolution media, was observed for celecoxib-PAA ASD, concomitant with the emergence of needle-shaped crystals. However, crystallization was not observed in the case of celecoxib ASD prepared with HPMCAS [81].

##### 3.2.1.5 Gel formation during dissolution

**Fig. 14:**
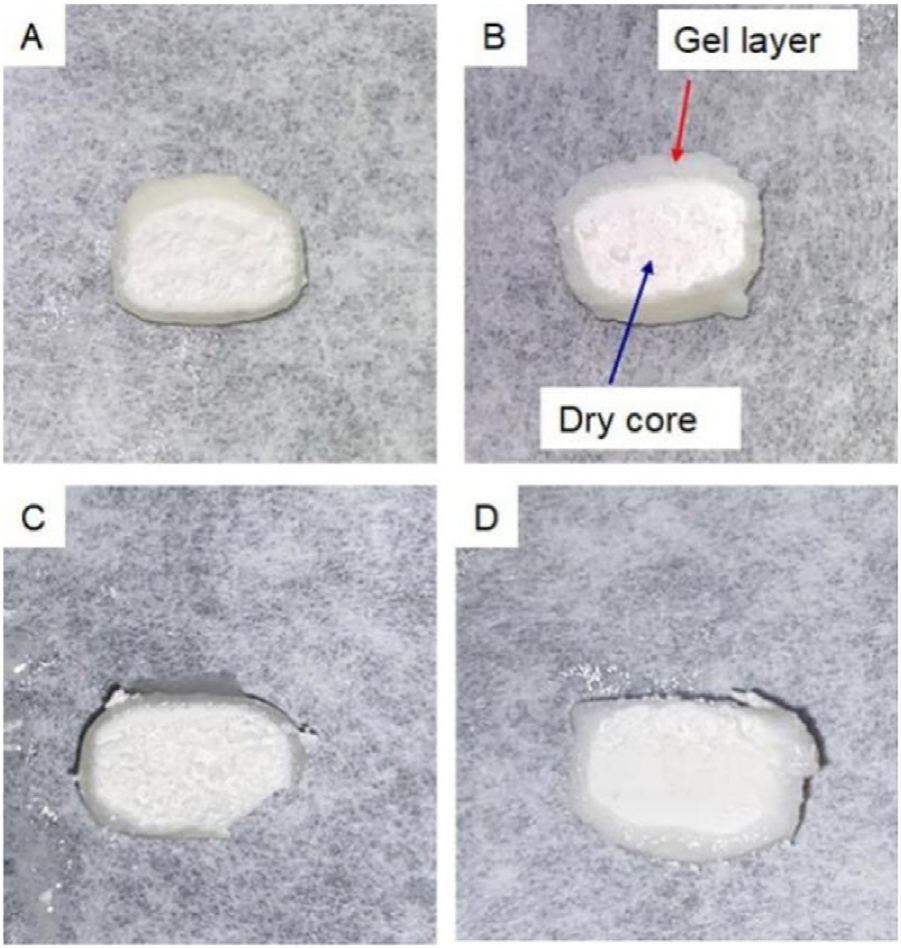
Gel layers formation and dry cores within tablets after exposure to a dissolution medium for approximately 10 min; (A) 40% neat PVPVA; (B) 40% PVPVA-drug 80:20; (C) 40% neat HPMC and (D) 40% HPMC-drug 80:20 . Adapted from [197] with the permission.

A gel layer may form on the ASD surface during dissolution and retard drug release. If a continuous hydrophobic phase is formed in the gel layer and if this network persisted over time, then the contents in the hydrophobic phase cannot release. Zhang et al. systematically evaluated the impacts of polymer type, polymer-drug ratio, and ASD loading on the dissolution [197]. The results showed that at high drug loadings, ASD tablets of HPMCAS exhibited faster drug release than other ASD of PVP/VA or HPMC. The slower drug release by hydrophilic polymer-based ASDs was caused by gel formation on the surfaces of the ASDs (Fig. 14) [197]. Similar slow dissolutions of tablets based on gel formation by the polymer were also reported in other studies [198], [199], [200]. Xi et al. found that gel layer during dissolution blocked water penetration to the tablet core and hence retarded tablet disintegration, while certain kosmotropic salts could significantly accelerate tablet disintegration [200]. Deac et al. used a fluorescence confocal microscopy for *in situ* monitoring of a gel layer and its phase behavior at the ASD/solution interface during dissolution [187]. Two model compounds, phenolphthalein and its methoxy derivative formulated as ASDs with PVPVA were investigated [187]. It was found that gel formation started with water penetration into the initially glassy solid, which plasticized the glass and caused a sharp glass-rubbery transition. As a result, the polymer chains had very slow translational diffusion and did not immediately release into the bulk aqueous media. Therefore, polymers would impact the gel formation due to their different water sorption abilities and different glass-rubbery transition points [187].

##### 3.2.1.6 Polymer-drug interactions

**Fig. 15:**
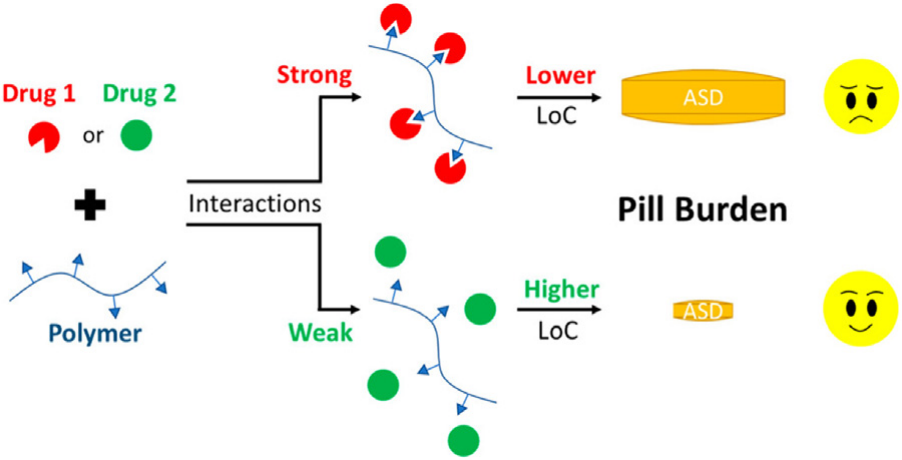
Schematic for the relationships between drug-polymer interactions and the LoCs for ASDs . Adapted from [201]with the permission.

The ideal dissolution profile is that the drug and the polymer are released with the same normalized rate [201]. The polymer-drug interactions may affect their congruent release from the ASD [184,[201], [202], [203], [204]. The maximal drug loading at which congruent release occurs is defined as the LoC [201,^204^,205]. Below the LoC, the drug and polymer percent release rates are similar, and in this congruent release regime, the drug and polymer release to completion. While at higher drug loadings above LoC, both drug and polymer release becomes incongruent and drug release is suppressed [187].Taylor et al. investigated the relationships between the drug chemical structures and the LoCs for PVP/VA-based ASDs [201,206]. They found that ASDs with stronger intermolecular interactions had lower LoCs, while drugs that formed weaker interactions with the polymer had considerably higher LoCs (Fig. 15) [201]. Recently, Hiew et al. compared the solid-state stabilities and release characteristics of ASDs containing lumefantrine when formulated with PVP/VA and four other polymers HPMCAS; HPMCP; CAP; Eudragit^Ⓡ^ L100 [184]. XPS data showed that lumefantrine exhibited acid-base interactions with the enteric polymers and formed ion pairs, and no interaction was observed for PVP/VA ASDs, which led to faster release than those seen for ASDs with enteric polymers [184]. The interaction between lumefantrine and the polymer was expected to retard polymer dissolution [184].

##### 3.2.1.7 Residual crystallinity

**Fig. 16:**
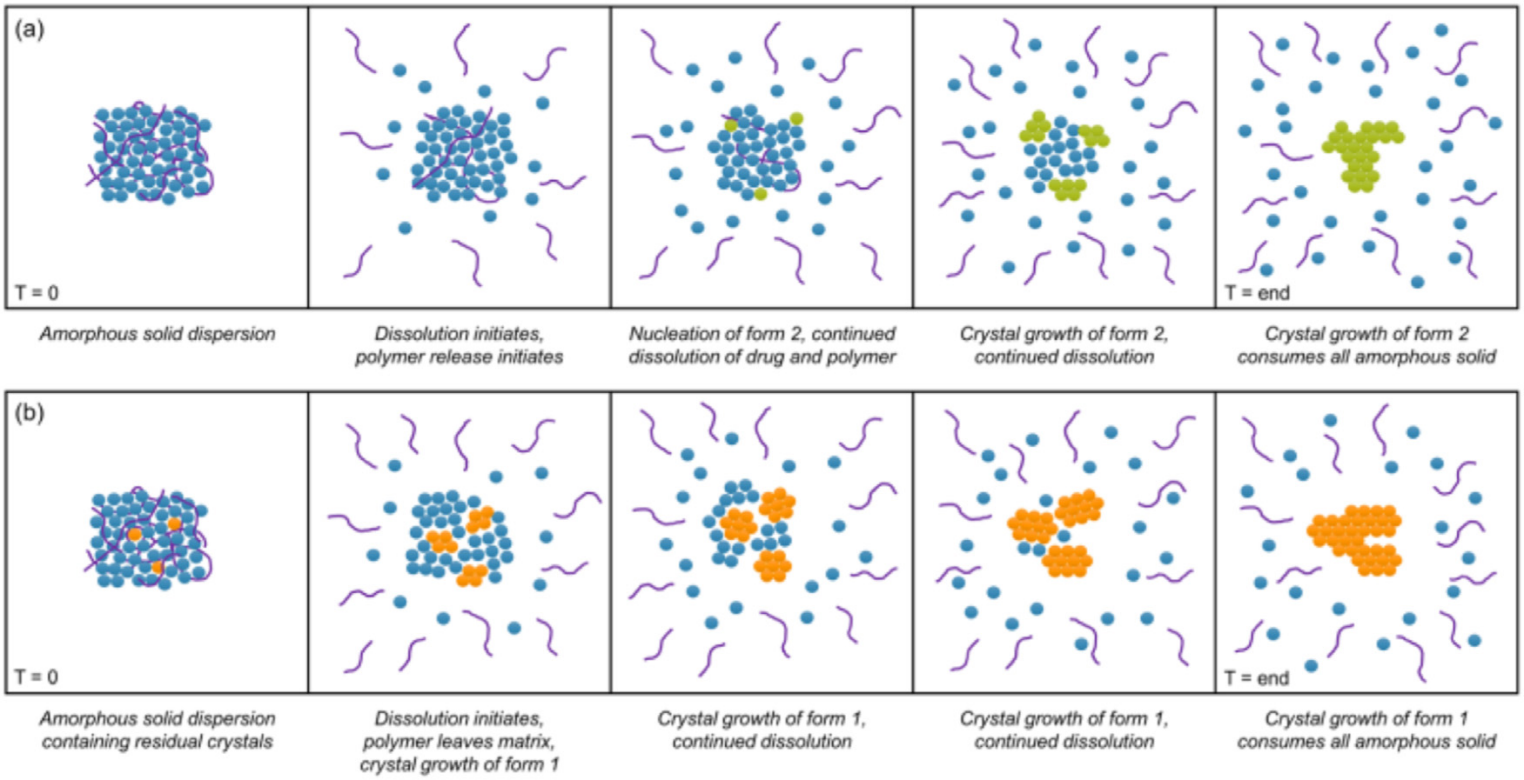
Schematic for the dissolution and crystallization for bicalutamide/PVPVA ASDs (a) without residual crystallinity and (b) with residual crystallinity. Adapted from [209]with the permission.

Residual crystallinity in an ASD may have a negative effect on the drug dissolution by directly reducing the solubility and leading to the loss of supersaturation [207,208]. Moseson et al. evaluated the impacts of residual crystallinity on dissolution performance of indomethacin/PVPVA ASDs [207]. They found that ASDs containing residual crystals lost the solubility advantage during non-sink conditions [207]. However, the presence of PVP/VA was found to stabilize the attained supersaturation by inhibiting crystallization [207]. Extensive adsorption of the polymers onto the residual crystals poisoned crystal growth, as evidenced by atomic force microscopy and scanning electron microscopy [207]. ASDs with residual crystallinity may undergo matrix crystallization rapidly during dissolution. Moseson et al. evaluated the effects of residual crystallinity within bicalutamide (BCL)/PVPVA ASDs on the underlying dissolution and crystallization [209]. The ASDs without residual crystallinity were not released completely and crystallized to form a metastable form (form 2) [209]. The ASDs with residual crystals had markedly reduced supersaturation. Crystallization consumed the amorphous drug and resulted in the stable form (form 1) (Fig. 16) [209]. Recently, Guner e et al. found that during the dissolution of griseofulvin ASDs, the residual nanocrystal seeds and the higher seed loading caused a faster desupersaturation than the micron-sized crystal seeds[208]. Soluplus^Ⓡ^ was a better nucleation and drug precipitation inhibitor than HPMC and PVP/VA due to its higher hydrophobicity [208].

#### 3.2.2 Maintaining the supersaturated state

Rapid dissolution of amorphous drugs to form a supersaturated solution in the gastrointestinal fluid leads to improved bioavailability [210]. It is also important to maintain supersaturation by inhibiting drug crystallization [210,211]. The rat jejunal perfusion assay demonstrated that supersaturated drug solutions enhanced the diffusion of drugs across biological membranes [211].

##### 3.2.2.1 Level of supersaturation

The level of supersaturation influences the stabilities of ASDs against crystallization when they are suspended in aqueous media [212].The release rates of ASDs may influence the supersaturation level and then impact the drug crystallization. Alonzo et al. reported that polymers became less effective in inhibiting drug crystallization as the supersaturation level increased [170]. For ASDs, fast dissolution may lead to a high degree of supersaturation and fast crystallization. Sun et al. reported that rapid generation of a highly supersaturated solution was not ideal for maintaining supersaturation since a high degree of supersaturation can accelerate crystallization in solution [213,214]. However, slow drug release led to a low initial degree of supersaturation and slow crystallization [213,214]. They proposed that a modest supersaturation generation rate was ideal in producing sufficiently high maximum kinetic solubility [213,214]. The gradual release of an ASD could prevent a sharp surge of supersaturation during dissolution, and thus, supersaturation could be sustained over an extended period without crystallization, which would improve the solubility and bioavailability [215].

##### 3.2.2.2 Polymer-drug interactions

**Fig. 17:**
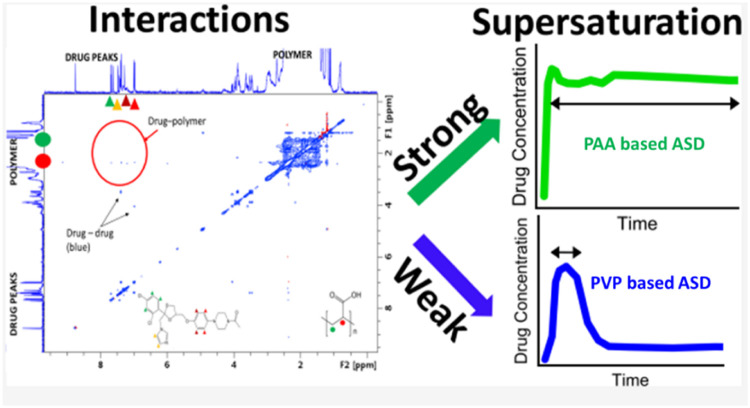
Schematic illustration of the interactions between ketoconazole and PAA, which were confirmed by two-dimensional ^1^H NOESY and the dissolution of KTZ ASDs formulated with PAA and PVP. Adapted from [218] with the permission.

The performance of ASDs in aqueous media is strongly impacted by the drug-polymer interactions [216], [217], [218]. Favorable drug-polymer interactions are important in maintaining supersaturation of the ASD during dissolution [217], [218], [219], [220], [221], [222]. Suryanarayanan and coworkers investigated the dissolution behaviors of ketoconazole (KTZ) ASDs [218]. The PAA-based ASD maintained supersaturation for a longer duration than the PVP-based ASD (Fig. 17) [218]. The drug-polymer interactions between ketoconazole and PAA were confirmed by two-dimensional ^1^H nuclear Overhauser effect spectroscopy (NOESY) (Fig. 16). Drug-polymer interactions were not detected in aqueous solutions [218].

The substituent levels in some polymers affect their ability to decrease crystallization rate and maintain supersaturation [219], [220], [221], [222]. Hu et al. compared the supersaturation kinetics of albendazole and the effects of various polymers, including PVP, PVP/VA, HPMC, and HPMCAS [222]. HPMCAS was most effective in inhibiting nucleation due to hydrophobic interactions of drug and succinoyl substituents in HPMCAS [222]. In a study of new cellulose derivatives and their abilities to inhibit crystallization from solution, moderately hydrophobic cellulose-based polymers with highly ionizable carboxylic acid substituents showed effective inhibition of crystallization [219], [220], [221]. Thus, in many studies, HPMC and HPMCAS were more effective than hydrophilic polymers in maintaining supersaturation [219,[223], [224], [225], [226]. For example, in the dissolution study of danazol ASDs with PVP, HPMC, and HPMCAS, the system containing PVP had the shortest crystallization induction times [223].

In addition, the drugs-polymers interactions in solution can be significantly influenced by surfactants [227,228]. Pui et al. reported that nimodipine formed hydrophobic interactions with PVP in the supersaturated solutions, and thus the supersaturation was prolonged significantly in the presence of PVP [227]. However, the addition of low-concentration SLS decreased the ability of PVP to maintain supersaturation due to the fact that SLS could disrupt the PVP-nimodipine interactions by competing with drugs to form a strong hydrophobic interaction with PVP [227].

##### 3.2.2.3 Adsorption of the polymer at the solid‒liquid interface

**Fig. 18:**
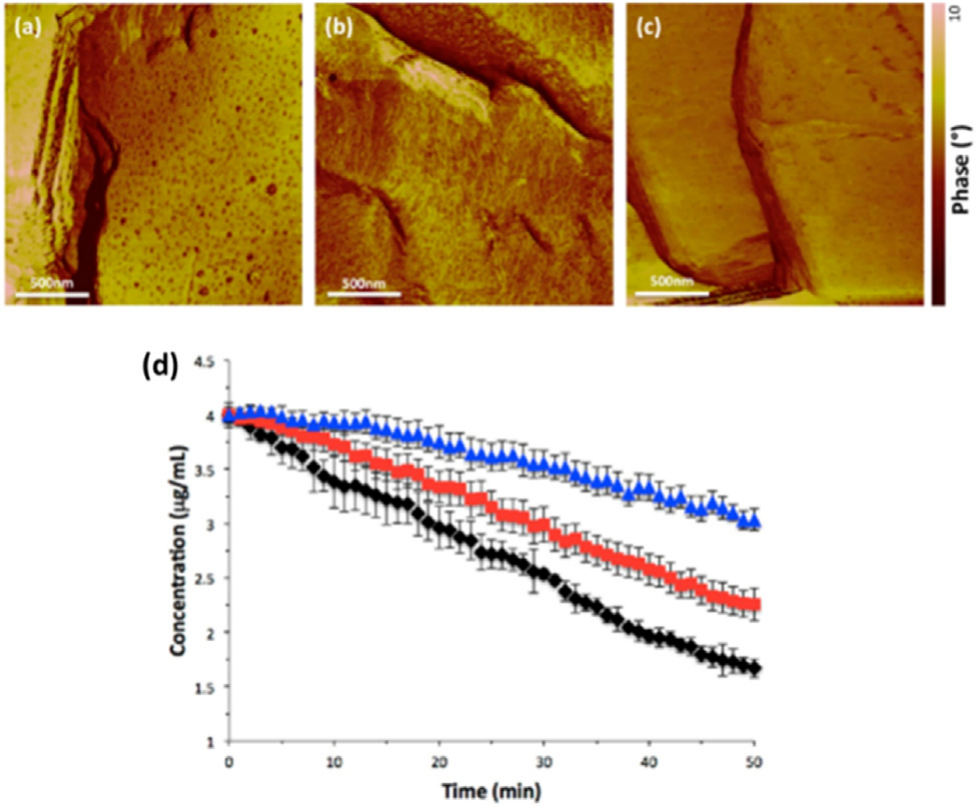
AFM images for HPMCAS adsorbed to felodipine (a) at pH 3, (b) pH 6.8, and (c) no HPMCAS adsorbed. (d) desupersaturation in the absence of HPMCAS (◆) and in the presence of HPMCAS at pH 3 (■) and pH 6.8 (▲) . Adapted from [231] with the permission.

The interactions between polymers and the surfaces of crystalline drugs could contribute to the inhibition of crystallization by blocking integration of the solute into the lattice [84,^224^,^229^,230]. For example, in a study of crystallization kinetics of felodipine in a solution containing HPMCAS, polymer adsorption on crystal surfaces decreased the felodipine crystal growth rate, as observed with atomic force microscopy (AFM) [230]. The adsorbed polymer conformation on a crystalline surface could affect the crystal growth in the aqueous phase [231]. Schram et al. used AFM to compare the conformations of HPMCAS adsorbed on felodipine at two pHs (Fig. 18) [231]. HPMCAS is insoluble in acidic aqueous media, but it dissolves when the pH is above 5.5 [14] . HPMCAS exhibits an extended chain conformation at pH 6.8 and forms compact coils at pH 3. As shown in Fig. 18a, the dark spots provide evidence for HPMCAS adsorbed at pH 3.At pH 6.8, the AFM revealed dark shading (Fig. 18b). The adsorbed HPMCAS provided a high degree of surface coverage and had a greater inhibitory effect on felodipine crystal growth at pH 6.8 (Fig. 18d) [231].

##### 3.2.2.4 Liquid‒liquid phase separation (LLPS)

**Fig. 19:**
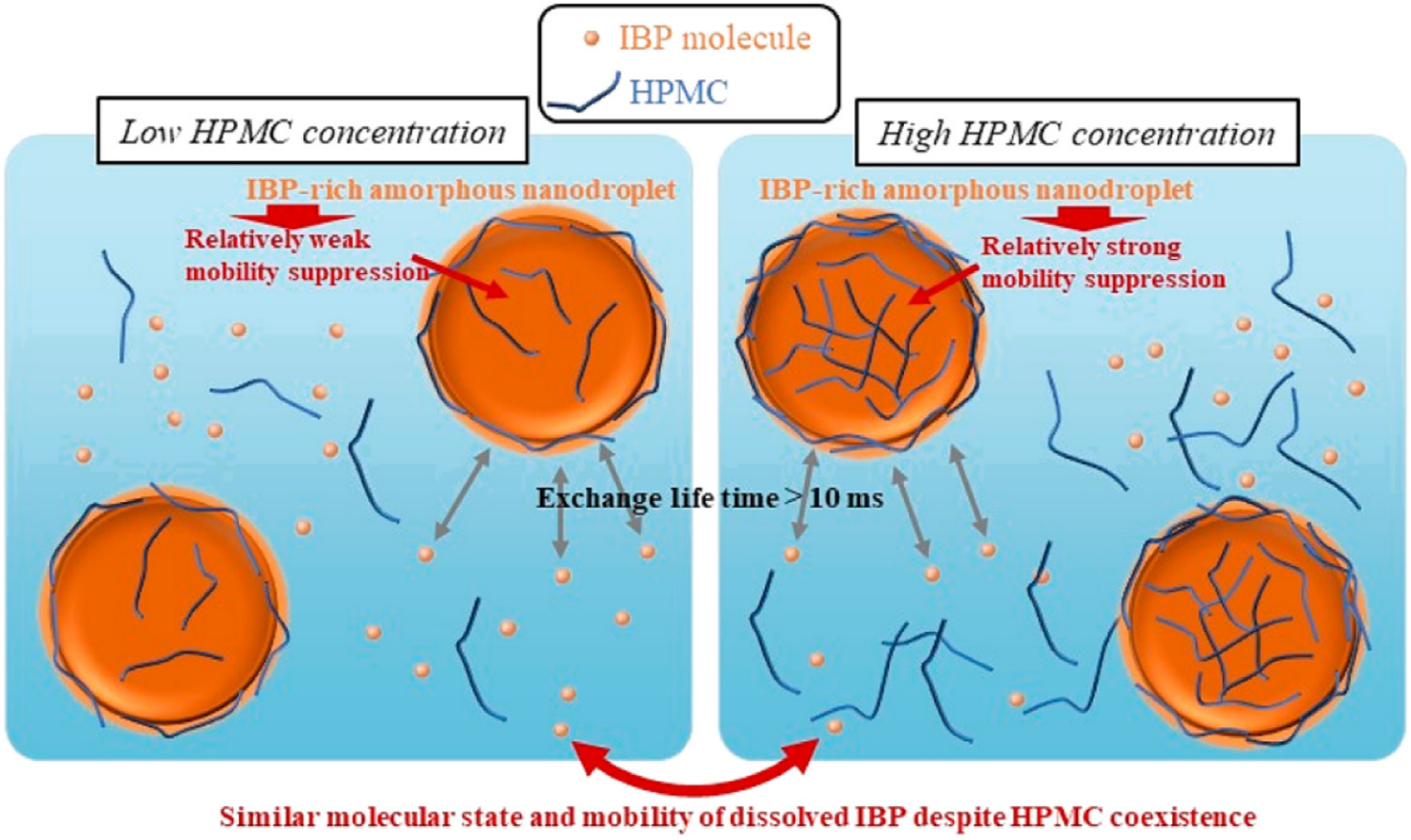
Schematic illustration of IBP-rich nanodroplets stabilized by HPMC in supersaturated solution. Adapted from [236] with the permission.

For poorly water-soluble drugs that crystallize slowly, when the amorphous solubility is exceeded during dissolution, the drugs undergo LLPS to form drug-rich nanodroplets (sometimes called drug-rich colloids) [14,^15^,232]. For LLPS, the drug-rich phase have sizes of 100–500 nm when detected immediately after formation with dynamic light scattering [232]. The stability of LLPS has been studied extensively and is related to the supersaturation state [232], [233], [234]. Taylor and coworkers demonstrated that the drug-rich species served as a reservoir to maintain the maximum free drug concentration [233,235]. The drug in the reservoir replenished the aqueous phase, thus maintaining the same activity after some of the drug diffused through the membrane [235]. Polymers play important roles in resisting drug crystallization and particle evolution, and they stabilize nanodroplets [236,237]. Ueda et al. reported that HPMC was distributed into ibuprofen (IBP)-rich nanodroplets [236]. The incorporation of the HPMC suppressed crystallization from the drug-rich species by altering the chemical environment of IBP-rich nanodroplets, which strongly decreased the molecular mobility (Fig. 19) [236]. A suitable polymer may stabilize the drug-rich nanodroplets and thus enhance drug absorption. Ueda and Taylor investigated the impacts of various polymers on drug solubility and stability of the IBP-rich amorphous nanodroplets [237]. The solution NMR spectroscopy indicated that large amounts of polymers were distributed into the drug-rich droplets [237]. Mixing of the polymers with the IBP-rich phase could decrease amorphous solubility and smaller droplet sizes [237]. The drug-rich droplets were more stable when HPMC was present due to steric repulsion from the HPMC adsorbed at the IBP droplet−water interface [237]. In contrast, Eudragit^Ⓡ^ E PO did not mix with the IBP-rich phase, which led to larger initial droplet sizes. However, Eudragit^Ⓡ^ E PO increased the solubility of amorphous IBP through drug-polymer complex formation in solution [237].

Surfactants added to ASD formulations may also influence the LLPS [238], [239], [240]. Taylor and coworkers evaluated the impacts of surfactants on the processability and release rates of clopidogrel ASDs. The nonionic surfactants partitioned into the drug-rich species, while the ionic surfactants interacted at the interface of nanodroplets [239]. Some types of surfactant restricted nanodroplet growth, but the surfactant cetrimonium bromide destabilized the nanodroplets [239].

The polymer and drug interactions can also influence the LLPS during dissolution. Qian and coworkers prepared three felodipine ASDs with PVP, PVP-VA, or HPMC-ASD [241].Rapid dissolution of the amorphous felodipine yielded LLPS, but the nanospecies obtained from the three ASDs were different [241]. For example, in 0.05 M HCl, the PVP/VA ASD showed rapid dissolution accompanied by nanospecies generation when the drug loading was less than 15%, while in the PVP system, only when the drug loading was less than 10%, the same phenomenon can be obtained and HPMCAS ASDs were released slowly without forming nanospecies [241]. The Flory-Huggins model showed that molecular interactions decreased in the order PVP/VA > PVP > HPMCAS [241]. Thus, the authors speculated that water-resistant drug-polymer interactions impact the formation of the nanospecies during dissolution [241].

### 3.3 Bioavailability

#### 3.3.1 In vitro/in vivo correlation

The ultimate success of an ASD is to increase bioavailability of poorly soluble drugs [242,243]. In general, passive permeation of drugs should increase with increasing concentrations of dissolved drug molecules [14,15]. The increased amounts of dissolved drugs in the gastrointestinal tract could lead to increase permeability and then result in enhanced absorption and bioavailability [14,^15^,^244^,245]. Metre et al. investigated ASDs comprising rivaroxaban with Eudragit^Ⓡ^ S100, Eudragit^Ⓡ^ L100, or Soluplus^Ⓡ^. Soluplus^Ⓡ^-based ASDs yielded the best dissolution and solubility for rivaroxaban and the best enhancement in oral bioavailability and C_max_ among all ASDs [58]. Similar observations were reported in a study of a silymarin ASD [28]. The PVP K17-based ASD significantly improved both the dissolution and bioavailability of silymarin [28]. *In vitro*, the cumulative amount of drug released from the silymarin powder was less than 30% after 120 min, while the PVP K17-based ASD showed the most rapid drug dissolution and a 98% cumulative drug release within 15 min [28]. *In vivo*, the *AUC* and the *C*_max_ of silymarin ASD were 2.4-fold and 1.9-fold higher than those of the pure drug, respectively [28].

However, it is often challenging to construct a good *in vitro*-*in vivo* correlation (IVIVC) because of complex nature of the *in vivo* process [242,246]. A few successful examples of IVIVC for ASD have been reported in the literature. The *in vivo* situation is more complex than those of *in vitro* media, so *in vitro* dissolution cannot be expected to represent the full picture for *in vivo* solubility and dissolution. For example, Sun and coworkers prepared an ASD comprising sorafenib (SOR) and HPMCAS by coprecipitation [247]. During the dissolution experiment, the ASD maintained a drug concentration of ∼60 μg/ml, while Nexavar^Ⓡ^ (a marketed product of SOR) led to a maximum concentration of ∼20 μg/ml. An *in vivo* pharmacokinetic study in dogs showed that ASD tablets increased near 50% higher bioavailabilities than Nexavar^Ⓡ^ [247]. Although the ASD tablets and Nexavar^Ⓡ^ have the same *in vitro* and *in vivo* performance, the relative improvement in bioavailability of the SOR ASD tablets was less than the vastly improved *in vitro* drug release [247]. ASDs with better drug release rates during *in vitro* dissolution may have lower oral bioavailability [248]. For instance, Six et al. evaluated the dissolution rates and oral bioavailabilities of ASDs containing itraconazole with HPMC, Eudragit^Ⓡ^ E100, or a mixture of Eudragit^Ⓡ^ E100 and PVP/VA [248]. They found that the ASD formulations with Eudragit^Ⓡ^ E100 or Eudragit^Ⓡ^ E100-PVP/VA exhibited more rapid rates of dissolution, but they had lower oral bioavailability [248]. The authors speculated that the poor predictability of bioavailability based on dissolution data was either due (i) to decreasing in drug solubility, (ii) to incomplete tablets disintegration, or (iii) to crystallization when ASD was granulated and tableted [248].

Many efforts have been done to demonstrate improved IVIVC. There are a number of studies that apply biorelevant media to represent environment on *in vivo* solubility and dissolution of ASDs to improve IVIVC [7,^14^,15]. Meanwhile, the biphasic test is also been employed to simulate the dynamic processes (*e.g.*, dissolution, precipitation, and partition) [249], Xu et al. evaluated three commercial products of ritonavir (the tablet, powder, and solution) by two biorelevant, and a “Level A” type of IVIVC is obtained [249]. Gao et al. applied machine learning to predict formulation composition, dissolution profiles and *in vivo* absorption behavior of ASDs to improve IVIVC. The computational methods could significantly facilitate pharmaceutical formulation development than the traditional trial-and-error approach. [250].

#### 3.3.2 Effects of polymers on permeability

The polymers may influence the membrane permeability of the drug in ASD. The improved bioavailability of ASDs can be reinforced by using a polymer that increases the permeability of the drug [251]. For instance, Shi et al. developed an ASD with berberine and HPC [251]. The results showed that the ASD dissolution rate was similar or even lower than that of the physical mixture. However, the relative bioavailability of ASD to its physical mixture was 303.0% [251]. They demonstrated that the berberine ASD increased the berberine concentration compared to crystalline drug, which lead to enhanced permeability [251]. Moreover, this drug is a P-gp (P-Glycoprotein) substrate; the authors supposed that the saturation of the P-gp because of amorphous berberine supersaturation improved intestinal absorption and bioavailability [251].

The polymers in ASDs may also promote permeability of drug by lowering the phospholipid bilayer order level [67]. Fan et al. elucidated the impact of using HPMC as an assistant excipient on improving membrane permeability in curcumin ASDs formulated with Eudragit^Ⓡ^ E100 [67]. The ability of HPMC to retard crystallization and improve membrane permeability was confirmed by fluorescence spectroscopy and permeability experiments [67]. The addition of HPMC to curcumin ASDs enhanced the drug permeability by lowering the order level of the phospholipid bilayer [67].

#### 3.3.3 Effects of surfactants

Surfactants have been reported to increase solubilities and dissolution rates of poorly soluble drugs [136,^252^,253]. In many studies, the use of surfactants in ASD formulations may affect *in vitro*/*in vivo* correlation [254,255]. Surfactants can act as wetting agents to enhance the access of water molecules to hydrophobic drugs. Meng et al. investigated the impact of D-α-tocopheryl polyethylene glycol 1000 succinate (vitamin E TPGS) on the wettability of celecoxib ASDs through contact angle determination [256]. The contact angle decreased as the level of TPGS increased in the formulations, proving that the presence of TPGS enhanced the wettability of the ASD with a faster dissolution rate [256]. The ternary ASDs prepared by ritonavir, PVP/VA and a surfactant (SDS, Tween 80, Span 20 or Span 85) showed that the drug release rates were improved at 30% DL for all surfactants. However, only SDS and Tween 80 improved drug release at higher DLs. They concluded that the SDS and Tween 80 in ASDs increased drug-polymer-water miscibility and disruption of the drug-rich barrier at the gel-solvent interface via plasticization [257]. If the surfactant content in the dissolution medium reaches or exceeds the critical micellar concentration (CMC), a significant impact on the dissolution behavior is expected as the formation of micelles by surfactants can increase drug solubility [256].

**Fig. 20:**
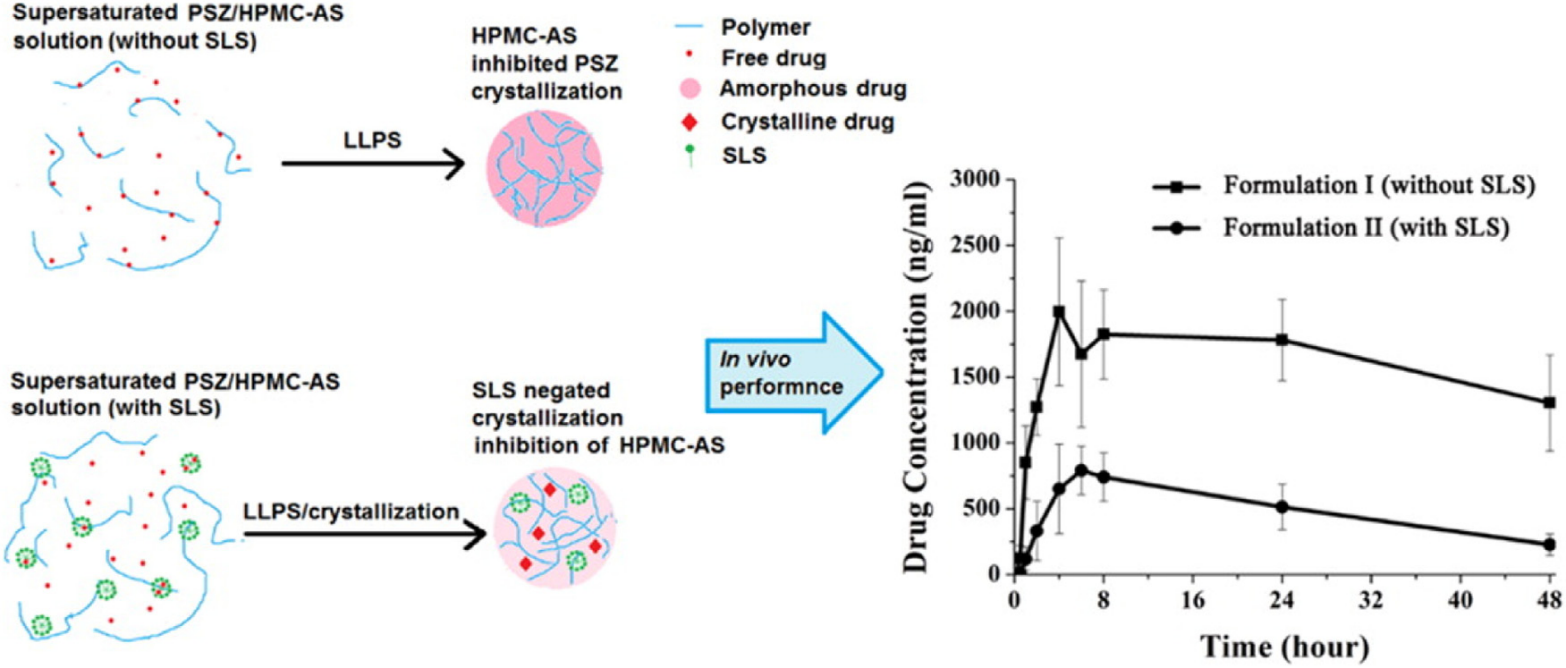
Schematic illustration of the impacts of SLS on dissolution and bioavailability of PSZ/HPMCAS ASDs. Adapted from [255] with the permission.

However, Chen et al. evaluated the impact of SLS on the dissolution and bioavailability of an ASD containing posaconazole and HPMCAS [254]. The formulation containing SLS showed a lower bioavailability than the SLS-free ASD formulation, despite better *in vitro* dissolution. *In vitro*, SLS increased solubility of drug and increased wetting of the ASD with SLS. However, surfactants such as SLS interact competitively with the polymer and thus might weaken the ability of the polymer to retard crystallization, leading to a decrease in bioavailability (Fig. 20) [254]. Similar observations reported by Tung et al. that addition of certain concentration of a surfactant (Poloxamer 188) in l-tetrahydropalmatine ASD below the critical micelle concentration induced precipitation during dissolution [255]. The 1.5% surfactant in ASD decreased the bioavailability by nearly 2.4 times [255]. Correa-Soto et al. investigated several ionic and nonionic surfactants and their effects on ASDs with high *T_g_* compounds. Without addition of surfactants, poor drug release was found for drug loadings above 5% due to the high *T_g_* of the ASD. While for several surfactants, drug release was enhanced. The *T_g_* of the ASD decreased upon addition of surfactants and water sorption extent increased during dissolution. The authors proposed that the surfactants acted as plasticizers, which facilitated polymer release and thus improved drug release. Thus the *T_g_* of the ASD, as well as the ability of the solvent to plasticize the system are important factors impacting the polymer release rate [258].

## 4 Manufacturing ASDs

A number of preparation methods have been developed for manufacturing ASDs [259]. Spray drying and HME have been widely used in manufacturing amorphous drugs on large scales. In recent years, some new technologies have been developed to manufacture ASDs, including the microwave method [260], 3D printing method [261], and solvent-based electrospinning method [181,262], etc.

The selection of a suitable preparation method is the crucial step in manufacturing robust ASD products [259]. The properties of both drugs and polymers influence the preparation methods and corresponding process parameters selected [259,260]. In this section, we will describe the influence of the polymer on the selection of a manufacturing method and the critical material attributes of polymers resulting from different manufacturing processes.

### 4.1 Spray drying

If a drug is thermally unstable, solvent-based methods are preferred for preparing ASDs. Commonly used solution methods, including spray drying, solvent evaporation, coprecipitation, and supercritical fluids, have been used to manufacture ASDs [15]. The critical material attributes of the polymers that should be considered include the polymer solvent solubility and feed solution viscosity.

Spray drying is the most widely used solvent-based method due to its solvent removal efficiency [4]. The spray drying is a continuous operation and can be realized easily across all scales from the lab to industry. Polymers such as PVP and HPMCAS are frequently applied in manufacturing ASDs by spray drying [259].

The challenge in developing an ASD by spray drying lies in finding a suitable solvent that solubilizes the drug and polymer to a sufficient extent. In general, the polymer and drug solubility is higher than 50 mg/mL which could achieve sufficient ASD producing efficiency [259]. From a Good Manufacturing Practice (GMP) perspective, the solvent should have low toxicity and high volatility [259]. The residual solvents in ASD must be within the acceptable limits of the International Conference Harmonization (ICH) guidelines [259]. These guidelines categorized solvents into three different classes, Class 1, 2 and 3. Class 3 is the least toxic solvent and is preferably used in preparing ASDs. It was suggested that the most toxic solvents, those in Class 1, should be avoided. However, selecting a suitable Class 3 solvent that dissolves both the drug and the polymer sufficiently with an acceptable viscosity is not always straightforward. Therefore, Class 2 solvents may be used in solvent-based processes for manufacturing ASDs [259]. Table 4 shows the polymers frequently used in approved ASD products and the commonly used solvents [263].

**Table 4:** Solubility of frequently used polymers of ASD in commonly used solvents [263].

Solvent	Copovidone	Povidone	HPMC	HPC	HPMCAS
Dichloromethane (DCM)	**S**	**S**	**I**	**S**	**I**
Acetone	**S**	**I**	**PS**	**I**	**S**
Methanol (MeOH)	**S**	**S**	**I**	**S**	**S**
Ethanol	**S**	**S**	**I**	**S**	**I**
Isopropanol	**S**	**S**	**I**	**S**	**I**
Tetrahydrofuran (THF)	**PS**	**I**	**I**	**S**	**S**
Ethyl acetate	**I**	**I**	**I**	**I**	**S**
2:1 DCM:MeOH	**S**	**S**	**PS**	**S**	**S**
2:1 Acetone:MeOH	**S**	**S**	**PS**	**S**	**S**
2:1 MeOH:THF	**S**	**S**	**I**	**S**	**S**

“S” is soluble (>5 w/w%), “PS” is partially soluble (1–5 w/w%), “I” is insoluble (<1 w/w%).

The viscosity of feed solution is also critical for the spray drying process [4]. Dispersed polymers are typically the main contributors to the spray solution viscosity [4]. The solution viscosity must be appropriate because it affects the feeding and drying processes [4]. Both of the solutions or suspensions can be used in the spray drying process, and high viscosity of feeding liquid may block the pressure nozzle [15].

The polymer properties, such as surface tension and diffusion rate, may also influence the spray drying process and microstructure of final ASD product. Chen et al. found that enrichment or depletion of the drug was detected at surface of ASDs prepared by spray drying [264]. They found that the relative surface tension and the strengths of intermolecular interactions might influence the surface composition of the ASD [264].

### 4.2 Melt extrusion

If the drug is thermally stable, fusion or melting methods may be used to prepare an ASD, including melt quenching, holt melt extrusion, and KinetiSol**^Ⓡ^** [15]. For these methods, the potential toxicity from residual solvent does not need to be considered because no solvent is involved in the process. However, these methods also have their own challenges. The polymers should be thermally stable and exhibit thermoplastic and thixotropic behavior.

HME is a well-known melting method and has been a topic of interest in manufacturing ASDs [265]. HME is advantageous over solvent-based methods because it is a solvent-free process [266]. Thus, it is a “green” process with fewer safety or environmental concerns [15]. Other advantages, such as continuous production of ASDs, are possible so that large-scale production can easily be achieved.

Recently, the nanoextrusion process has been developed to disperse drug nanoparticles in polymeric carriers using the conventional HME equipment [267,268]. Li et al. prepared nanocomposites of griseofulvin by nanoextrusion method [267]. The nanocrystals suspension was prepared by wet-milling along with the stabilizers HPC and Soluplus^Ⓡ^, and then the nanoextrusion applied the drug suspension as a feed, along with additional polymer (HPC/Soluplus^Ⓡ^) to disperse drug nanoparticles in the polymeric matrix, yielding the extrudates of nanocomposites [267].

The molten polymer must be miscible with the drug [15]. The changes in *T*_m_ and *T*_g_ as a function of drug loading can establish a phase diagram with which to aid the selection of a manufacturing temperature [15]. Tian et al. constructed thermodynamic phase diagrams for ASD systems and utilized them to guide HME process [269]. They found that this approach ensured the desired quality of ASDs [269].The type of polymers can affect the miscibility and homogeneity of ASD prepared by HME [270]. Kramarczyk et al. studied the impact of four model polymers (Kollidon^Ⓡ^ K30, Kollidon^Ⓡ^ VA64, Parteck MXP and Kollicoat IR) on the physicochemical properties of the posaconazole ASD systems manufactured by HME [270]. The increase of amorphousness of the polymer enhanced the physical stability of the ASDs. Compared to homopolymers, the ASDs formulated with copolymers exhibited greater homogeneity but less enhancement in aqueous solubility [270].

The polymer should have both thermostability and mechanostability during the manufacturing process [271]. The high temperatures and mechanical stresses produced in the manufacturing process may cause polymer and drug degradation [271]. The processing temperature and mechanical stress should be appropriate to allow practical manufacturing without the potential for drug degradation [272].

The most important physical property of the polymers used in the melting method is the melt viscosity. During the manufacturing process, the heated and possibly sheared liquid should flow through the heated barrel and out of the die without causing excessive torque on the electric motor. The HME process should be operated at a temperature at least 20 °C above *T*_m_ of the semicrystalline polymer (or drug) or the *T*_g_ of the amorphous polymeric carrier [14]. Moreover, an excessively low-viscosity liquid has difficulty maintaining its shape when it runs out of the die [273]. The complex viscosity of a neat polymer suitable for the extrusion operation is between 10,000 and 800 Pa^.^s [273,274].

Plasticizers can be applied in melting process to decrease manufacturing temperature or the viscosity of the formulation and thus facilitate thermal processing and melt extrusion [15]. It was reported that maximum temperature reductions as high as 50 °C were observed with Kollidon^Ⓡ^ SR containing a plasticizer, such as Lutrol^Ⓡ^ F68, Cremophor^Ⓡ^ RH40 and PEG 1500, during the HME process [274]. Meanwhile, drug itself can act as a plasticizer to reduce the process temperature [275,276]. For example, Repka et al. evaluated the application of efavirenz as a plasticizer for Eudragit^Ⓡ^ E PO or Plasdone S-630-based ASDs [277].

### 4.3 Other methods

New technologies have been developed in recent years to produce ASDs, including milling/cryogrinding, microwave treatments, 3D printing, solvent-based electrospinning, etc.

#### 4.3.1 Milling/cryogrinding

When a crystalline compound is subjected to high-energy milling, its crystal structure and microstructural characteristics are changed significantly. Reductions in the particle sizes have been known to reduce the crystallinity and result in amorphization. Milling/cryogrinding has been widely used for producing ASDs on a laboratory scale [278,279]. The choice of polymer affects the preparation of amorphous drugs with the milling method. Mesallati et al. prepared ciprofloxacin ASDs by ball milling; they found that acidic polymer was necessary to produce ASDs, as it enabled the formation of ionic interactions of drugs and polymers [280].

#### 4.3.2 Microwave

Microwave irradiation and the oscillating electromagnetic field apply friction to dipolar molecules, and thus, heat is generated [281]. Microwave-induced amorphization has been investigated with microwave ovens, wherein the crystalline drug is amorphized *in situ*. A luteolin ASD was prepared by the methods including fusion (FU), solvent evaporation (SE), and microwave irradiation (MI). The best dissolution enhancement for luteolin was achieved with an ASD of a luteolin:PEG 4000(ratio of 1:2) ASD prepared by the MI method [282]. In general, the drugs and polymers in ASDs are usually poor microwave absorbers and are hardly heated due to their low or moderate dielectric loss factors; thus, dielectric-enabling excipients, such as water or glycerol, are usually added. Water is critical for ASD preparation via the microwave method due to its dipolar nature, and it is heated when exposed to electromagnetic waves [283]. Qiang investigated the effects of polymers on microwave-induced amorphization of the indomethacin-polymer-water system. They found that the polymer Mw and the *T_g_* of the moisture-plasticized polymer, played crucial roles in microwave method [260]. It was shown that the water-polymer interactions were important for amorphization; the loosely bound water generated heat and evaporated, whereas the tightly bound water contributed less to heat generation and was less likely to evaporate [260].

#### 4.3.3 Three-dimensional (3D) printing

3D printing is an innovative technology used to transform 3D computer models into physical objects by additive manufacturing [284]. It is also one of the fastest developing technologies within the pharmaceutical manufacturing field [284]. Jamróz et al. explored the impacts of the dual coextrusion process on the properties of 3D-printed aripiprazole tablets. A ZMorph^Ⓡ^ 3D printer combined with a DualPro extruder was applied to produce the tablets, and the amorphization of aripiprazole was confirmed with X-ray diffraction patterns [284]. Santitewagun et al. applied terahertz spectroscopy to evaluate the crystallinities of ASDs produced with 3D printing and selective laser sintering. They noted that there was no mixing of the components during the selective laser sintering 3D printing process, which led to partially amorphous systems exhibiting poor reproducibility and physical stability problems [285]. Parulski et al. prepared an itraconazole ASD by using HME coupled with fused deposition modeling. This method enabled production of solid oral forms containing ASDs with different infill densities, which improved the drug release rate [286].

3D printing has often been applied in combination with HME to prepare ASD; thus, stability and a suitable viscosity of the polymer for processing are crucial physicochemical properties [287]. In terms of polymer selection for 3D printing, a number of suitable carrier materials have been reported in the literature, such as PVA, poly(lactic acid) (PLA), Eudragit^Ⓡ^ E PO, and Soluplus^Ⓡ^ [3,^288^,289].

#### 4.3.4 Electrospinning

Electrospinning generates nanofiber-like ASDs from solutions, melts under high voltage electric fields [3,^290^,291]. It has many advantages over other methods, such as ease of implementation and generation of ASDs with large surface areas and high porosities [3,^290^,291]. Becelaere et al. produced stable nanofibrous flubendazole ASDs with 2-ethyl-2-oxazoline and the solvent electrospinning method. The ASDs showed ultrahigh drug loadings (up to 55% drug) and long-term stabilities (at least one year) [292]. Moreover, the large specific surface areas and high porosities of the nanofibrous nonwovens enhanced the dissolution rates of the ASDs [292].

Balogh et al. applied melt electrospinning to prepare carvedilol ASDs. The drug carvedilol and the polymer Eudragit^Ⓡ^ E, a third component, plasticizer was added, which effectively lowered the viscosity and manufacturing temperature of the melt process. The dissolution results showed that plasticizers accelerated dissolution [293]. Almost all polymers used in ASD manufacturing can be used for electrospinning, including PVP, PEO, PVA, HPMC, and the Eudragit^Ⓡ^ series [291]. The type of polymer should be considered when preparing the electrospinning fluid. All fluids containing drugs and polymers, such as those used in solution, melt and melt-solution processing, should have appropriate viscosities [290].

## 5 Safety considerations and regulatory approval of polymeric excipients

### 5.1 Safety of the polymer

Polymers are integral components of ASD formulations, and they should be nontoxic and pharmacologically inert [14,15]. The polymeric carrier should be a pharmaceutical-grade polymer, as these are labelled “generally regarded as safe” (GRAS) [2]. In the FDA inactive ingredient database, a list of safe polymer excipients and their safe content levels are provided [2]. Despite the variety of the available polymers, it is better to employ polymeric excipients that have proven safety. Safe polymeric excipients and their safe percentage levels are determined based on prior use in existing products or must be justified with the proper preclinical and clinical data [263]. Table 5 shows the commonly used polymer excipients approved by all major regulatory authorities (FDA, EMA and PMDA) or monographs published in the relevant pharmacopoeias (USP, Ph. Eur., JP) [14]. New polymer excipients have been developed in many studies [220,[294], [295], [296], [297]. However, new polymers in ASDs intended for human use require extensive evaluation and review with a battery of standard tests for pharmacological activity, chronic toxicity and carcinogenicity [2,^14^,15].

**Table 5:** Safety information of common polymers used in ASD [14].

Polymer type	Structural features	IID(Inactive Ingredient Database) limits	LD50	Regulatory status
PVP	neutral	80 mg	>100 g/kg	USP/Ph.Eur/JPE
PVP/VA	neutral	853.8 mg	>10,000 mg/kg	USP/Ph.Eur/JPE
Soluplus^Ⓡ^	neutral	N/A	>5000 mg/kg	N/A
HPC	neutral	240 mg	>10.2 g/kg	USP/Ph.Eur/JPE
HPMC	neutral	480 mg	>4000 mg/kg/day	USP/Ph.Eur/JPE
HPMCAS	acidic	560 mg	>2.5 g/kg	USP/Ph.Eur/JPE
Eudragit^Ⓡ^ EPO	alkaline	10 mg	N/A	USP/Ph.Eur/JPE
Eudragit^Ⓡ^ L100	acidic	93.36 mg	Rat >15,900 mg/kg Mouse>10,000 mg/kg Dog >10,000 mg/kg	USP/Ph.Eur/JPE
Eudragit^Ⓡ^ L100–55	acidic	99.99 mg	N/A	USP/Ph.Eur/JPE

### 5.2 Quality by design (QbD)

The development process for formulations follows the Quality by Design (QbD) strategy recommended by global regulatory authorities [14,15]. The QbD principles were laid out in ICH document Q8 [14,15].

There are a number of literatures that apply QbD in the development of ASD formulations using robust and reliable processes [277,^298^,299]. QbD begins with a quality target product profile (QTPP). Then, QbD can be used to define the critical quality attributes (CQAs). Physical stability and dissolution are very important CQAs for an ASD. These CQAs identify and link the critical material attributes (CMAs) and process parameters (CPPs) to the QTPP. Finally, risk assessments, experimental design, prior experience/knowledge, and literature are used in defining and controlling the design space.

The selection of an appropriate polymer is the key to obtain an ASD formulation exhibiting satisfactory performance. The physicochemical properties of the polymers are one of the determinants of the CQAs for ASDs. The CMAs of a polymer are the polymer type, molecular weight, polydispersity, *T*_g_, particle size, hygroscopicity, mechanical properties, chemical stability, etc. [2]. The impacts of the polymer properties on the process parameters and crystallization tendency of the final product should be understood. In early formulation development, it is critical to monitor the physical stability of an ASD during storage, usage and dissolution testing. This is one of the key regulatory focuses on the ASD development to ensure the therapeutical efficacy of the final product. The availability and use of various analytical techniques are essential for ensuring ASD quality throughout development stage. PXRD is the most commonly used technique, with other techniques such as DSC, PLM, Raman spectroscopy, FT-IR spectroscopy, dielectric spectroscopy, and NMR spectroscopy used to provide supporting evidence to confirm the amorphous state of the product [263].

## 6 Conclusion

**Fig. 21:**
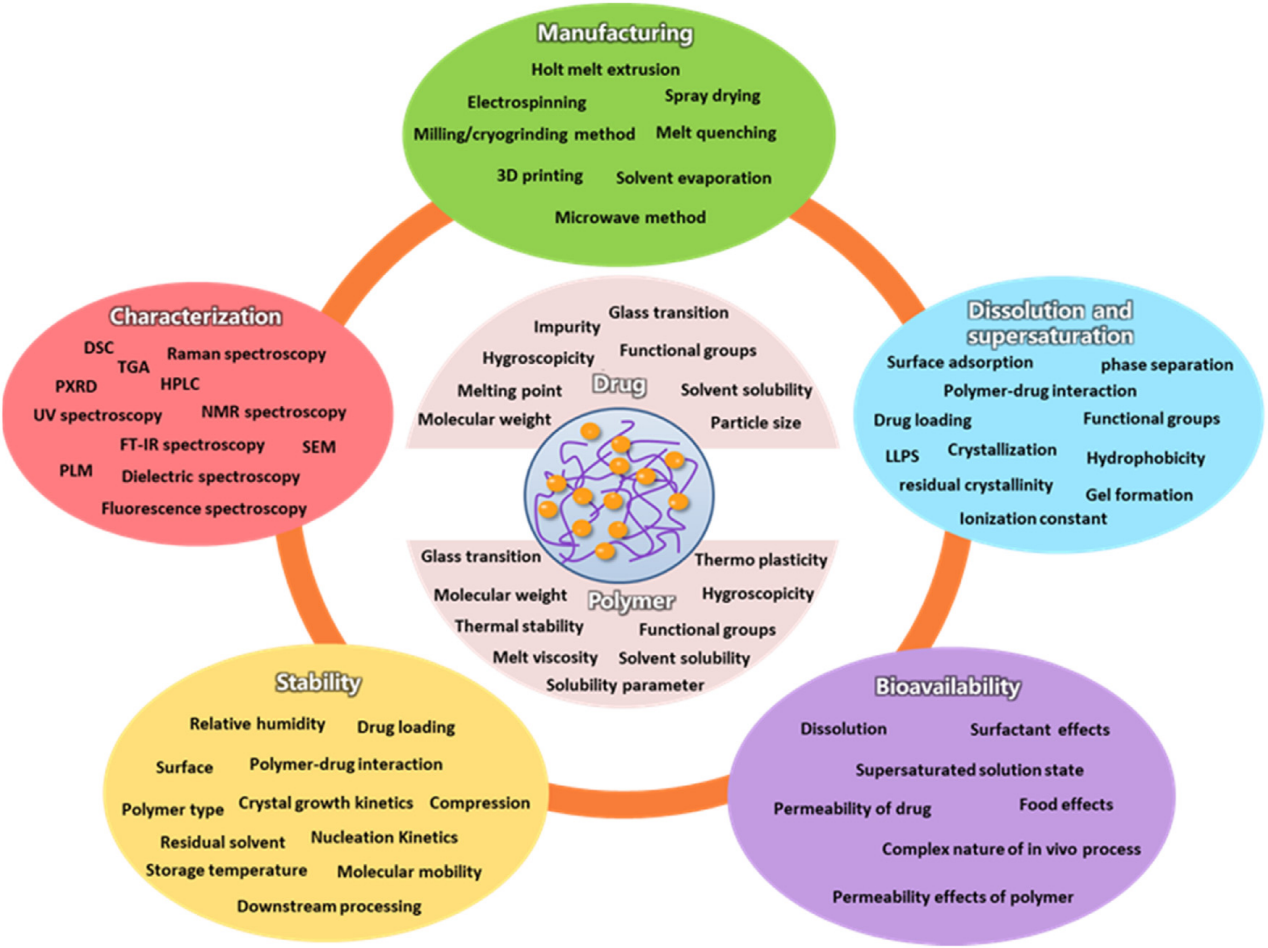
Detailed considerations for ASD formulation development.

In the past decades, the rapidly growing number of new drug compounds with low aqueous solubility presents serious challenges in the development of new pharmaceutical products. ASD is an established and effective formulation approach to increase the aqueous solubility and bioavailability of poorly soluble compounds. Polymers in the ASDs play critical roles in stabilizing amorphous drugs, maintaining supersaturated solutions, and achieving the desired bioavailability. The physicochemical properties of polymers should be taken into consideration when designing the formulation, selecting the manufacturing method, and pursuing safety/regulatory approvals for ASDs. Detailed information and mechanisms are needed to understand the critical drug and polymer attributes, critical material attributes and process parameters, and these are presented in Fig. 21 as examples.

Recently, it was reported that one polymer could combine with another polymer to enhance the performance of an ASD [66,^68^,^72^,245]. The use of multiple stabilizers in ASDs has been initiated to improve solubilization or stabilization of the ASD. The trends for ASD development are shifting toward the use of a second or even more polymers in addition to the primary polymeric carrier. It is worth mentioning that a binary or ternary mixture has the potential to combine the performance characteristics of different polymers.

Artificial intelligence (AI) is rapidly opening a new frontier in ASD development. Neural networks and deep learning methods have gained significant interest. Deep learning-based prediction methods have been applied to predict physical stabilities of ASDs, and this could replace the long times and high costs of conventional ASD stability tests [300]. Machine learning integrated with molecular dynamic (MD) simulation and physiologically based pharmacokinetic (PBPK) modeling have been developed to predict formulation composition, dissolution profiles and *in vivo* absorption behavior of ASDs [250]. In the future, the computational methods may show more potential in selecting the polymers suitable for ASD preparation than conventional approaches. Meanwhile, AI may also be applied to design and synthesize new polymers that are suitable for ASD formulations.

Numerous articles have described the syntheses, characterization data and applications of novel synthetic polymers for the development of ASDs. The new polymer-based ASDs may exhibit better *in vitro* or *in vivo* performance. However, the new polymers will require extensive testing and evaluation to ensure safe use in human pharmaceuticals without chronic toxicity or carcinogenicity.

## Conflicts of interest

The authors declare that there is no conflicts of interest.
